# Identification of Genes Involved in Biogenesis of Outer Membrane Vesicles (OMVs) in *Salmonella enterica* Serovar Typhi

**DOI:** 10.3389/fmicb.2019.00104

**Published:** 2019-02-04

**Authors:** Jan Nevermann, Andrés Silva, Carolina Otero, Diego P. Oyarzún, Boris Barrera, Fernando Gil, Iván L. Calderón, Juan A. Fuentes

**Affiliations:** ^1^Laboratorio de Genética y Patogénesis Bacteriana, Facultad de Ciencias de la Vida, Universidad Andres Bello, Santiago, Chile; ^2^Escuela de Química y Farmacia, Facultad de Medicina, Universidad Andres Bello, Santiago, Chile; ^3^Center of Applied Nanosciences, Universidad Andres Bello, Santiago, Chile; ^4^Unidad de Microbiología, Hospital Clínico Universidad de Chile, Santiago, Chile; ^5^Microbiota-Host Interactions and Clostridia Research Group, Facultad de Ciencias de la Vida, Universidad Andres Bello, Santiago, Chile

**Keywords:** Outer membrane vesicles, *Salmonella* Typhi, *ompA*, *mrcB*, *rfaE*, *yibP*, *tolR*, *degS*

## Abstract

Outer membrane vesicles (OMVs) are nano-sized proteoliposomes discharged from the cell envelope of Gram-negative bacteria. OMVs normally contain toxins, enzymes and other factors, and are used as vehicles in a process that has been considered a generalized, evolutionarily conserved delivery system among bacteria. Furthermore, OMVs can be used in biotechnological applications that require delivery of biomolecules, such as vaccines, remarking the importance of their study. Although it is known that *Salmonella enterica* serovar Typhi (*S.* Typhi), the etiological agent of typhoid fever in humans, delivers toxins (e.g., HlyE) via OMVs, there are no reports identifying genetic determinants of the OMV biogenesis in this serovar. In the present work, and with the aim to identify genes participating in OMV biogenesis in *S.* Typhi, we screened 15,000 random insertion mutants for increased HlyE secretion. We found 9 *S.* Typhi genes (generically called *zzz* genes) determining an increased HlyE secretion that were also involved in OMV biogenesis. The genes corresponded to *ompA, nlpI*, and *tolR* (envelope stability), *rfaE* and *waaC* (LPS synthesis), *yipP* (*envC*), *mrcB* (synthesis and remodeling of peptidoglycan), *degS* (stress sensor serine endopeptidase) and *hns* (global transcriptional regulator). We found that *S.* Typhi Δ*zzz* mutants were prone to secrete periplasmic, functional proteins with a relatively good envelope integrity. In addition, we showed that zzz genes participate in OMV biogenesis, modulating different properties such as OMV size distribution, OMV yield and OMV protein cargo.

## Introduction

Outer membrane vesicles (OMVs) are nano-sized proteoliposomes (20–200 nm) discharged from the cell envelope of Gram-negative bacteria in a process that does not involve cell lysis or death (Schwechheimer and Kuehn, 2015). OMVs normally contain toxins, enzymes and other factors, and are used as vehicles in a process that has been considered a generalized, evolutionarily conserved delivery system among bacteria (Schwechheimer and Kuehn, 2015). In pathogenic bacteria, OMVs can be directly delivered into host cells during infection. Many virulence factors are secreted via this pathway, such as the vacuolating toxin VacA of *Helicobacter pylori* and the HlyE hemolysin in *Salmonella enterica* serovar Typhi (*S.* Typhi) (Wai et al., 2003; Ricci et al., 2005). On the other hand, and due to their immunogenicity and ability to display antigens, OMVs can be incorporated into vaccine preparations. Since OMVs are metabolically inert, they represent fewer risks compared with live-cell vaccines (van der Pol et al., 2015). Nevertheless, increasing protective responses generated by OMVs, engineering the inclusion of protective antigens, and reducing OMV-mediated toxicity remain challenges in this field (van der Pol et al., 2015).

Evidence indicates that OMV biogenesis relies on three main mechanisms: (1) dissociation of the outer membrane in specific zones lacking proper attachments to underlying structures (e.g., peptidoglycan) (Yeh et al., 2010; Park et al., 2012); (2) the presence of misfolded proteins, which accumulates in nanoterritories where crosslinks between peptidoglycan and other components of bacterial envelope are either locally depleted or displaced (Schwechheimer and Kuehn, 2015); and (3) some changes in LPS composition also modulate OMV biogenesis, presumably by generating a differential curvature, fluidity, and/or charge in the outer membrane (Elhenawy et al., 2016). In this sense, identification of genes involved in such processes has been progressively gaining attention.

Global incidence of typhoid fever, a severe disease produced by *S.* Typhi (*Enterobacteriaceae*), is estimated to cause over 21 million cases worldwide, with approximately 220,000 deaths each year (Crump et al., 2008). The full progression of this disease was commonly observed in the pre-antibiotic era, resulting in 30% mortality. Antibiotics greatly reduced mortality, but emergence of multi-resistant strains represents a serious issue. Thus, a deeper knowledge of *S.* Typhi pathogenesis and the molecular determinants of the progression toward a systemic infection are needed to develop new approaches to treat or prevent typhoid fever in the future. At present, OMVs biogenesis is poorly understood in *Salmonella enterica*, and unknown in *S.* Typhi. Studies performed in *S. enterica* serovar Typhimurium (*S.* Typhimurium) reveal that some changes in LPS composition, observed in Δ*pagL* mutants (lipid A deacylase), negatively affects OMV biogenesis (Elhenawy et al., 2016). Nevertheless, it is necessary to be cautious before directly extrapolating data from *S.* Typhimurium to *S.* Typhi, a common practice. There are differences in biogenesis, composition and activity of OMVs between species, presumably due to the particular ecological niche of each pathogen (McBroom and Kuehn, 2007). *S.* Typhi and *S.* Typhimurium, albeit closely related, exhibit considerable differences regarding host range and disease progression. While *S.* Typhi infects only humans, producing a systemic infection, *S.* Typhimurium infects a broad range of hosts, producing only a self-limited gastroenteritis in humans (Parkhill et al., 2001). Remarkably, *pagL* is a pseudogene (i.e., a non-functional gene) in *S.* Typhi (Parkhill et al., 2001). For that reason, data obtained from *S.* Typhimurium (and much less from *Escherichia coli*) cannot be directly extrapolated to *S.* Typhi without an experimental approach (Urrutia et al., 2014).

As stated, identifying genes involved in OMV biogenesis is the first approach to understand mechanisms potentially involved in modulating OMV properties, such as size distribution and protein cargo selection. Nevertheless, such identification is challenging. In *S.* Typhi, OMVs proved to be the delivery mechanism for at least two virulence factors: the typhoid toxin and the HlyE cytolysin (Wai et al., 2003; Guidi et al., 2013). Remarkably, these toxins are not produced by *S.* Typhimurium (McClelland et al., 2000; Fuentes et al., 2008), reinforcing the fact that an experimental strategy must be performed to better understand the role of OMVs in *S.* Typhi. In *S.* Typhi, HlyE (ClyA) is a periplasmic hemolysin that contributes to invasion of epithelial cells (Fuentes et al., 2008). Although *hlyE* is expressed under standard growth conditions, *S.* Typhi WT is not hemolytic on blood agar (Fuentes et al., 2008). Previously, we reported that *S.* Typhi Δ*ompA* exhibits hemolysis on blood agar due to an increased secretion of HlyE (Fuentes et al., 2008). Since OmpA participates in OMVs biogenesis in other *Enterobacteriaceae* (Deatherage et al., 2009), we hypothesized that *S.* Typhi Δ*ompA* presents an increased HlyE secretion due to changes in OMV production. In this sense, we reasoned that *S.* Typhi derivatives exhibiting increased HlyE secretion, evidenced by hemolysis on blood agar under standard growth conditions, will present changes in OMV biogenesis. In the present work, and with the aim to identify genes participating in OMV biogenesis in *S.* Typhi, we screened 15,000 random insertion mutants for increased HlyE secretion. We found 9 *S.* Typhi genes (generically called *zzz* genes) involved in OMV biogenesis, corresponding to *ompA, nlpI*, and *tolR* (envelope stability), *rfaE* and *waaC* (LPS synthesis), *yipP* (also known as *envC*), *mrcB* (synthesis and remodeling of peptidoglycan), *degS* (stress sensor serine endopeptidase) and *hns* (global transcriptional regulator). All these genes encode functions related to envelope stability, accumulation of misfolded proteins, or to LPS composition. In general, *S.* Typhi Δ*zzz* mutants were found to present an increased production of OMVs, which exhibited a distinct size distribution and protein cargo compared with the OMVs derived from the WT, supporting the role of *zzz* gene in OMV biogenesis in *S.* Typhi.

## Materials and Methods

### Bacterial Strains, Media and Culture Conditions

*Salmonella enterica* serovar Typhi strain STH2370 (*S.* Typhi WT) (Valenzuela et al., 2014) was obtained from the Infectious Diseases Hospital Lucio Córdova, Chile. *S. enterica* serovar Typhimurium ATCC14028s (*S.* Typhimurium WT) was obtained from the Instituto de Salud Pública, Chile. Strains were routinely grown in liquid culture using Luria Bertani medium (Bacto peptone, 10 g/L; Bacto yeast extract, 5 g/L; NaCl, 5 g/L; prepared in phosphate buffer pH 7.0) at 37°C, with aeration. When required, medium was supplemented with kanamycin (50 mg/ml), chloramphenicol (20 mg/ml), ampicillin (50 mg/ml), Xgal (5-bromo-4-chloro-3-indolyl-β-D-galactopyranoside) (40 μg/ml), or agar (15 g/L).

### Random Insertional Mutagenesis

Mutagenesis was performed using the EZ-Tn5 < R6Kγ*ori*/KAN-2 > transposome (Epicenter^®^) as previously described (see detailed description in Supplementary Figure 1) (Goryshin et al., 2000). We screened 15,000 mutants to cover all the genome, and hemolytic colonies were selected for further analyses. As the next step, a “clean mutagenesis” (backcross) of the corresponding gene interrupted by the transposon (i.e., the *zzz* gene) was performed in a *S.* Typhi WT background using Red-Swap recombination (Datsenko and Wanner, 2000) yielding *S.* Typhi Δ*zzz*::FRT mutants. For the experiments presented in this work, at least two different *S.* Typhi Δzzz clones, independently generated, were tested in each case. All mutants were corroborated by PCR amplification, as described (Datsenko and Wanner, 2000). *S.* Typhi Δ*zzz*::FRT Δ*hlyE*::kan double mutants were constructed using the Red/Swap method (Datsenko and Wanner, 2000) iteratively.

*S.* Typhi *hlyE*-3 × FLAG was constructed as previously described (Fuentes et al., 2009). *S.* Typhi Δ*zzz*::FRT *hlyE*-3 × FLAG was constructed by moving the *hlyE*-3 × FLAG (kan^R^) allele over the corresponding *S.* Typhi Δ*zzz*::FRT mutant as described (Toro et al., 1998). A full list with the primers used in this study is presented in the Supplementary Table 1.

To complement the *S.* Typhi Δ*zzz* mutants, we amplified the corresponding zzz genes (using primers listed in Supplementary Table 1) prior to cloning them into the pCR TOPO TA^®^ plasmid according to the manufacturer’s instructions.

### Quantitative RT-PCR Assay

Quantitative RT-PCR was performed as described (Jofre et al., 2014). Briefly, total mRNA from the strains grown in LB broth (approximately OD_600_ = 1.4), was extracted using TRIzol reagent (Invitrogen) as described by the manufacturer. RNA was precipitated with isopropanol for 10 min at room temperature, washed with ice-cold 70% v/v ethanol and resuspended in DEPC-treated water, prior to the treatment with DNase I to remove any trace of DNA. Purity of extracted RNA was determined by spectrometry. Reverse transcription and relative quantification of each mRNA was performed as previously described (Jofre et al., 2014). Experiments were performed in three biological and technical triplicates. Statistical significance of differences in the relative expression data was determined using Kruskal–Wallis with Dunn’s test as *post hoc* analysis to compare with the WT. A full list with the primers used in this study is presented in the Supplementary Table 1.

### Immunodetection Analysis and SDS–PAGE

Strains carrying the epitope-tagged *hlyE* gene were grown in LB at 37°C with shaking to stationary phase (approximately OD_600_ = 1.1). For the whole lysate, bacterial pellets were resuspended in 1 mL of Tris–HCl pH 8.0 and sonicated on ice during 100 s. For the supernatant fraction, proteins were previously precipitated using 1/4 volume of trichloroacetic acid, incubated at 4°C for 10 min, and centrifuged at 15,000 ×*g* for 10 min. The pellet was washed twice with cold acetone and finally resuspended in Tris–HCl 100 mM pH 8.0. Bradford method (Bradford, 1976) was used to calculate the volume of the whole lysate containing 60 μg of proteins for each strain to be tested (load control). An equivalent volume was used for the corresponding supernatant fraction. Proteins were resolved by 12% SDS–PAGE, transferred to poly(vinylidene difluoride) membranes and stained with Ponceau S to confirm the protein load as previously described (Stochaj et al., 2006). Western blot was performed as described, using Hsp60 as load control (Jofre et al., 2014; Ortega et al., 2016).

The same procedure described for the SDS–PAGE was used to qualitatively assess the exported proteins of *S.* Typhi WT and *S.* Typhi Δ*zzz* mutants.

In the case of OMV proteins, OMVs obtained from either *S.* Typhi WT or *S.* Typhi Δ*zzz* mutants (corresponding to 12 μg of proteins) were resolved by SDS–PAGE and visualized with silver stain.

### Bioassay for Determining β-Lactamase Release

First, strains expressing β-lactamase (*bla* gene) from a single chromosomal copy were constructed by the chromosomal insertion of pNFB9. pNFB9 is a plasmid that contains the *bla* gene, the attachment site for the phage Gifsy-1 (att*_PG1_*) and the respective integrase *int* (Lemire et al., 2008), allowing its integration into the att*_BG1_* chromosomal site of *S.* Typhi (naturally empty) (Valenzuela et al., 2014), yielding the strain *S.* Typhi att*_BG1_*::pNFB9 (Lac^-^ Amp^R^). The same procedure was performed for all the mutants (i.e., *S.* Typhi Δ*zzz*) to generate the corresponding Amp^R^ derivatives. Spots of *S.* Typhi *att_BG1_*::pNFB9 (WT background), and *S.* Typhi Δ*zzz att_BG1_*::pNFB9 derivatives (Δ*zzz* backgrounds) were plated over a lawn of *S.* Typhimurium ATCC14028s Δ*ompD*::Mu*d*J (10^6^ cfu, Lac^+^ Amp^S^, reporter strain) previously spread on a LB agar plate supplemented with ampicillin and Xgal. For semi-quantitative assay to assess export of β-lactamase, supernatants from the strains described above was obtained (OD_600_ = 1.1), filtered (0.45 μm), precipitated with trichloroacetic acid (as described above), serially diluted and used for the bioassay. In all cases, export of functional β-lactamase was inferred by the growth of blue satellite colonies.

### Assay of Resistance to Deoxycholic Acid and Vancomycin

Bacteria were grown in LB with shaking at 37°C to OD_600_ = 0.5 or 1.1, prior to being treated during 2 h with sodium deoxycholate 0.5% or PBS (control) at 37°C. Percentage of survival was calculated as following: (cfu treated with sodium deoxycholate/cfu with PBS) × 100, relative to *S.* Typhi WT. To determine sensitivity to vancomycin, a Kirby-Bauer assay was performed as reported (Villagra et al., 2012), using vancomycin disks loaded with 30 μg of antibiotic.

### Preparation of Bacterial Samples for Atomic Force Microscopy (AFM)

Preparation of bacterial samples was performed as described (Azari et al., 2013) with modifications. Briefly, bacterial suspensions (OD_600_ = 1.1) were centrifuged and washed three times with PBS. Aliquots were applied onto the surface of circular coverslips and allowed to dry. Bacterial cell surfaces were imaged in contact mode or optical fiber cantilevers [n-type silicon cantilevers (f 1/4 37.2 kHz; k 1/4 0.01–0.60 N/m; tip radius of 10 nm)], using a Nanonics MultiView MV1000. Analysis was performed with the Gwyddion 2.42 software.

### Determination of β-Galactosidase Release

*S.* Typhi WT and *S.* Typhi Δzzz derivatives were transformed with a religated pCR TOPO TA^®^ multicopy plasmid (Lac^+^), producing strains constitutively expressing β-galactosidase (since *S.* Typhi lacks the *lacI* gene) (Valenzuela et al., 2014). All the strains were tested in LB plates supplemented with ampicillin and Xgal before the assays. For the assay, bacteria were cultured in LB to approximately OD_600_ 1.1, prior to obtaining the pellet and the supernatant fraction by centrifugation. β-galactosidase activity was determined as described (Fuentes et al., 2009). The percentage of β-galactosidase release was calculated as (β-galactosidase activity of supernatant)/[(β-galactosidase activity of supernatant + β-galactosidase activity of pellet)] × 100.

### OMV Isolation, Quantification and Size Measurement

To isolate OMVs, we followed a previously reported protocol, with modifications (Liu et al., 2016b). Bacteria were grown in LB at 37°C with shaking (approximately to OD_600_ = 1.1), prior being centrifuged 10 min at 5400 ×*g* at 4°C. The pellet was discarded, and the supernatant fraction was filtered (0.45 μm), ultrafiltered with Ultracel^®^ 100 kDa ultrafiltration disks (Amicon Bioseparations), and ultracentrifuged 3 h at 150000 ×*g* at 4°C (Thermo Scientific™ Sorvall™ WX, Rotor AH-629). The supernatant was discarded, and the pellet resuspended in 1 ml DPBS. OMVs were stored at -20°C until their use. We quantified OMV yield as by determining the both protein content (BCA assay) and lipid content (FM4-64 molecular probe), and normalizing by CFU/ml (McBroom et al., 2006; Deatherage et al., 2009). We determined OMV size as described (Deatherage et al., 2009). Briefly, OMV size (diameter) was measured from at least 3 TEMs of 3 independent OMV extracts per strain in Adobe Photoshop using the ruler tool. Results were presented in diameter ranges of 3 nm, where 3 represents 1 to 3 nm. All OMVs larger than 60 nm were grouped in the last category (60+ nm).

### Transmission Electronic Microscopy

Outer membrane vesicle extracts were bound to formvar-coated slot grids, stained with 1% aqueous uranyl acetate for 1 min, and viewed with a Philips Tecnai 12 (Biotwin) transmission electron microscope.

### MALDI-TOF

Mass spectrometry based on Matrix Assisted Laser Desorption Ionization Time-of-Flight (MALDI-TOF) was performed in a VITEK^®^ MS device (bioMérieux) (wavelength: 337.1 nm, beam divergence: 3.5 × 3 mrad; rate repetition and pulse duration: 3 ns/60 Hz; Maximum output: 150 μJ/pulse). Data were processed by Acquisition Software. Experiments were performed in biological duplicate, using two independent OMV extracts.

## Results

### Generation of *S.* Typhi Hemolytic Mutants by Random Insertional Mutagenesis

**FIGURE 1:**
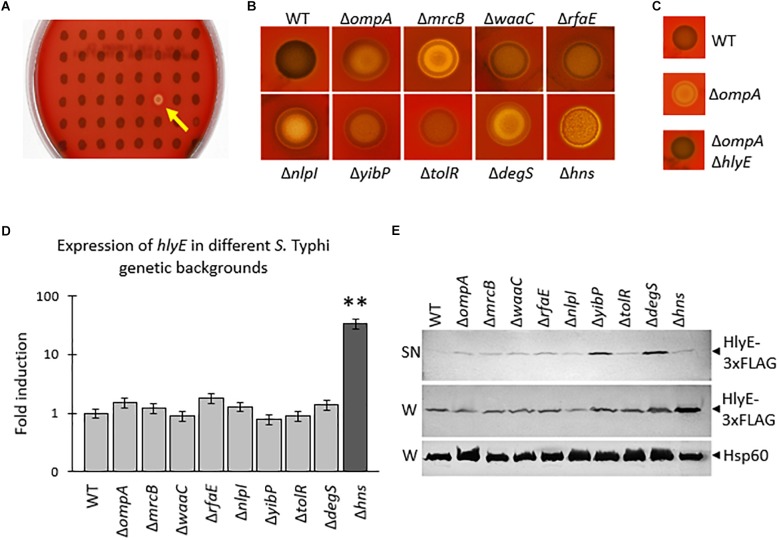
**(A)** Screening showing a hemolytic derivative (yellow arrow) found after random mutagenesis with Tn5. **(B)** Hemolysis on blood agar plates of *Salmonella enterica* serovar Typhi (*S.* Typhi) Δzzz::FRT mutants generated by Red-Swap recombination (Datsenko and Wanner, 2000). The WT and the mutants (stationary phase, 5 μl) were placed over blood agar plates and incubated during 12 h to reveal hemolysis. **(C)** The hemolysis depends on the presence of *hlyE*. All the *S.* Typhi Δ*zzz*::FRT Δ*hlyE*::kan double mutants lost the hemolytic phenotype, demonstrating that HlyE is the responsible of such phenotype. In the figure we show an example with *S.* Typhi WT, *S.* Typhi Δ*ompA*::FRT, and *S.* Typhi Δ*ompA*::FRT Δ*hlyE*::kan. The WT and the mutants were placed (stationary phase, 5 μl) over blood agar plates and incubated during 12 h to reveal hemolysis. **(D)** qRT-PCR to determine the *hlyE* expression in each mutant. In all the cases, the experiments were performed with 2 different clones for each mutant, in both technical and biological triplicate. Dark gray bar shows positive (^∗∗^*p* < 0.01) statistically significant differences compared with *S.* Typhi WT (*n* = 3 for each case, Kruskal–Wallis with Dunn’s test as *post hoc* analysis). **(E)** Secretion of HlyE to the supernatant fraction. *S.* Typhi *hlyE*-3 × FLAG (WT background) and *S.* Typhi Δ*zzz*::FRT *hlyE*-3 × FLAG mutants (Δ*zzz* background, indicated with the corresponding name of the deleted gene in the figure) were used to perform WB to detect HlyE-3 × FLAG in the supernatant (SN) or in the whole lysate (W). Detection of Hsp60 was performed as load control. In all cases, bacteria were grown in LB to stationary phase. This is a representative experiment of *n* = 5.

Although the cytolysin/hemolysin *hlyE* is efficiently expressed in rich media at 37°C, *S.* Typhi WT exhibits no hemolysis when cultured on blood agar plates (Jofre et al., 2014). Taking advantage of this feature, we performed a random insertional mutagenesis based on the EZ-Tn5 < R6Kγori/KAN-2 > transposome (Supplementary Figure 1) to find *S.* Typhi mutants with an increased HlyE secretion. To this purpose, we screened 15,000 random mutants (approximately 3 times the total number of *S.* Typhi genes, to cover the whole genome) (Porwollik et al., 2004; Valenzuela et al., 2014) on blood agar plates. Twenty-three clearly hemolytic colonies were identified and used for further analyses, including the identification of the corresponding interrupted genes (Figure 1A and Table 1). To corroborate that the observed hemolytic phenotype was effectively due to the loss of function of the identified genes, we performed a “clean” deletion by Red-Swap recombination (Datsenko and Wanner, 2000) for each identified gene in a *S.* Typhi WT background prior to testing the hemolytic phenotype of the resulting mutants. Some “clean” deletion mutants showed no hemolysis (Table 1), strongly suggesting that the hemolytic phenotype could be due to a secondary, spontaneous mutation or to polar effects attributed to the presence of the transposon. In these cases, genes were discarded. By contrast, 9 clean mutants (collectively called *S.* Typhi Δ*zzz*::FRT) conserved the hemolytic phenotype and were used for further analysis (Figure 1B and Supplementary Figure 2).

**Table 1:** Hemolytic *S.* Typhi mutants found after the screening^a^.

			Hemolysis observed in blood agar	
Interrupted gene	Function	Times found in the screening	EZ-Tn5^b^	Red-Swap^c^
*ompA*	Envelope stability	1	++	++
*mrcB*	Peptidoglycan synthesis	6	+++	+++
*waaC*	LPS synthesis	2	++	++
*rfaE*	LPS synthesis	2	++	++
*nlpI*	Envelope stability	1	+++	+++
*yibP* (*envC*)	Cell division	1	+	+
*tolR*	Envelope stability	1	+	+
*degS*	RpoE activator	1	+++	+++
*hns*	Global regulator	4	++++	++++
*rfbH^∗^*	Synthesis of antigen O	2	+	–
*ftsK^∗^*	Cell division	1	++	–
*ynhG^∗^*	Peptidoglycan assembly	1	+++	–

*^a^15,000 mutants were screened. ^b^Hemolytic phenotype observed after the random mutagenesis. ^c^Hemolytic phenotype observed after Red-Swap mutagenesis (backcross). ^∗^Hemolytic phenotype was not observed after Red-Swap mutagenesis (backcross).*

To determine that the observed hemolysis depends on the presence of *hlyE*, we generated *S.* Typhi Δ*zzz*::FRT Δ*hlyE*::kan double mutants. We were unable to detect hemolysis in the double mutants, showing that *hlyE* was effectively the genetic determinant of this phenotype (Figure 1C and Supplementary Figure 2).

To assess whether the Δ*zzz* deletions affected *hlyE* transcription, we performed qRT-PCR. As shown in Figure 1D, Δ*zzz* deletions did not affect *hlyE* transcription, indicating that the hemolytic phenotype exhibited by the *S.* Typhi Δ*zzz* mutants is more likely due to an increased HlyE secretion than to an increased *hlyE* expression. The only exception was observed with *S.* Typhi Δ*hns*, where the *hlyE* transcript was highly abundant compared with the WT, showing that H-NS contributes to the repression of *hlyE* expression (Figure 1D).

To corroborate that Δ*zzz* deletions contribute to an increased HlyE secretion, we epitope-tagged HlyE (Jofre et al., 2014), prior to analyzing the supernatant and the whole bacterial lysate by Western Blot. As shown in Figure 1E, almost all the mutants exhibited similar amounts of HlyE in the whole lysate. The increased production of HlyE, detected in the Δ*hns* genetic background, can be attributed to the increased amount of *hlyE* mRNA in this same background (Figure 1D). On the other hand, the presence of HlyE in the supernatant fraction, as an indicator of HlyE secretion, was clearly increased in all the tested mutants in comparison with the WT (Figure 1E).

Altogether, these results indicated that deletions of *zzz* genes produced an increased HlyE liberation.

### Δ*zzz* Deletions Are Involved in Releasing Distinct, Functional Proteins

**FIGURE 2:**
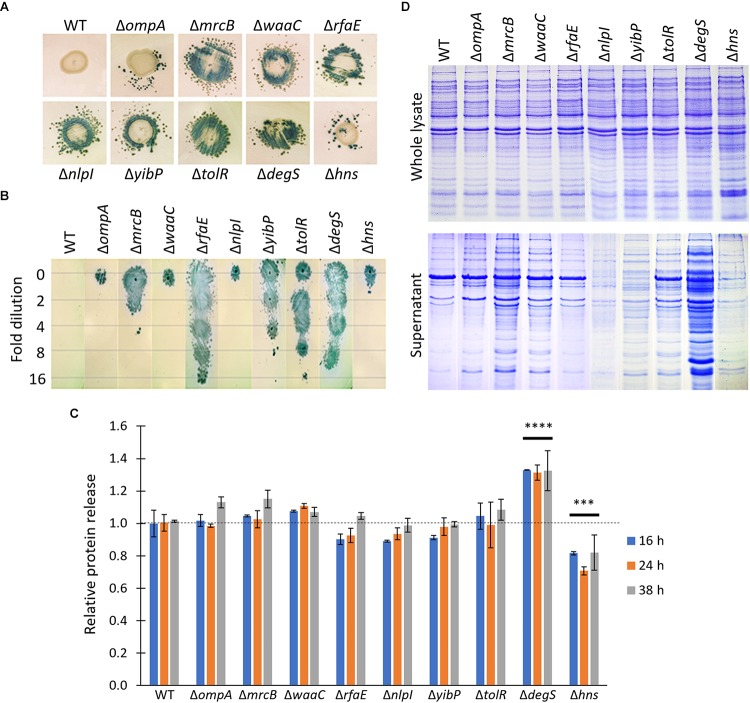
Δ*zzz* mutants release a distinct pattern of proteins. **(A)** *S.* Typhi Δ*att*_BG1_::pNFB9 (Lac^-^, and Amp^R^ due to the presence of *bla* gene stably inserted in the chromosome in one copy, WT background) and *S.* Typhi Δ*att*_BG1_::pNFB9 Δ*zzz*::FRT double mutants (Lac^-^ Amp^R^; Δ*zzz* background, indicated with the corresponding name of the deleted gene in the figure) were plated (stationary phase, 5 μl) over a lawn of a reporter strain (Lac^+^ Amp^S^) previously spread on a plate with Xgal and ampicillin. Release of functional β-lactamase was evidenced as the presence of blue, satellite colonies after 8 h of incubation. This is a representative experiment of *n* = 3. **(B)** Semi-quantitative assay to assess the export of periplasmic proteins (i.e., β-lactamase). Supernatant obtained from the same strains described in **(A)** was obtained and filtered (0.45 μm). Proteins were precipitated with trichloroacetic acid, serially diluted, and plated (5 μl) over a lawn of a reporter strain, as indicated in **(A)**. This is a representative experiment of *n* = 3. **(C)** SDS–PAGE showing proteins exported into the supernatant from *S.* Typhi WT and *S.* Typhi Δ*zzz* mutants. Bacteria were cultured in LB to stationary phase, prior to obtaining proteins from the supernatant fraction as described in the Section “Materials and Methods.” Proteins from the whole lysate were used as load control. We performed this same assay with two different clones for each mutant, obtaining similar results. This is a representative experiment of *n* = 5. **(D)** Protein release of WT and *S.* Typhi Δ*zzz* mutants. *S.* Typhi WT or *S.* Typhi Δ*zzz* mutants were cultured in LB with shaking at 37°C for 16, 24, or 38 h (approximately OD_600_ in each case = 1.1, stationary phase). Then, bacteria were centrifuged (15,000 × *g* for 5 min) to obtain the pellet and the supernatant fractions. Supernatant was filtered (0.45 μm) and proteins were precipitated with trichloroacetic acid as described in Materials and Methods. Protein content was determined for both fractions to calculate the % of protein release as (proteins in the supernatant fraction)/[(proteins in the supernatant fraction)+(proteins in the pellet fraction)]. In all cases, values were corrected by OD_600_. In the figure, we showed relative values to the WT at 16 h as reference. In each case, 2 independent clones were tested, with both technical and biological triplicate. Two-way ANOVA with Tukey as *post hoc* test was performed (^∗∗∗^*p* < 0.001 and ^∗∗∗∗^*p* < 0.0001). No statistically significant differences were found among 16, 24, and 38 h for each mutant.

Since periplasmic proteins are prone to be secreted via OMVs (Haurat et al., 2011), increased secretion of periplasmic, functional proteins, such as β-lactamase, has been considered a typical feature of hypervesiculating mutants (Schwechheimer and Kuehn, 2015). To determine whether Δ*zzz* deletions promote the release of periplasmic proteins, different from HlyE, we constructed a *S.* Typhi parental strain harboring one copy of the *bla* (β-lactamase) gene stably inserted into the chromosome (i.e., *S.* Typhi Δ*att*_BG1_::pNFB9). We used this strain to construct the *S.* Typhi Δ*att*_BG1_::pNFB9 Δ*zzz* double mutants. To determine whether Δ*zzz* deletions improve β-lactamase secretion, we performed a satellite-based bioassay. As shown in Figure 2A, all the *S.* Typhi Δ*att*_BG1_::pNFB9 Δ*zzz* mutants allowed the growth of blue, satellite colonies around a spot of bacteria plated over a lawn of a Lac^+^ Amp^S^ reporter strain. By contrast, no satellites were observed with the *S.* Typhi Δ*att*_BG1_::pNFB9 strain (WT background) (Figure 2A). As a semi-quantitative approximation, dilutions of precipitated proteins obtained from the corresponding supernatants were tested as described above. As shown in Figure 2B, some Δ*zzz* mutants seem to secrete higher amounts of β-lactamase, since it was possible to observe blue satellites even in the most diluted samples (e.g., Δ*rfaE*, Δ*tolR*, and Δ*degS*), whereas other *S.* Typhi Δ*zzz* mutants only allowed the growth of satellites with the most concentrated samples (e.g., Δ*ompA*, Δ*waaC*, Δ*nlpI*, and Δ*hns*). On the other hand, *S.* Typhi Δ*att*_BG1_::pNFB9 (WT background) supernatant was unable to allow the growth of satellites, even in the most concentrated sample. These results strongly suggest that Δ*zzz* mutants release distinct amounts of proteins. To test this hypothesis, we compared the amount of total released proteins from *S.* Typhi WT and *S.* Typhi Δ*zzz* mutants. We found that *S.* Typhi Δ*degS* secreted 30% more proteins into supernatant fraction compared with the WT (Figure 2C). This result might explain the increased secretion of both HlyE and β-lactamase in this mutant. Nevertheless, we observed that *S.* Typhi Δ*ompA*, Δ*mrcB*, Δ*waaC*, Δ*rfaE*, Δ*nlpI*, Δ*yibP*, and Δ*tolR* were undistinguishable from the WT regarding the amount of total secreted proteins (Figure 2C). On the other hand, *S.* Typhi Δ*hns* released approximately 25% fewer proteins (Figure 2C). These data suggest that the increased secretion of HlyE and β-lactamase cannot be merely explained by a generalized protein hypersecretion, at least in these last cases, suggesting that periplasmic proteins are prone to be secreted due to another phenomenon. These results prompted us to qualitatively assess the exported proteins by SDS–PAGE. As control, we loaded proteins obtained from the respective whole lysate. As shown in Figure 2D, *S.* Typhi Δ*zzz* mutants seem to secrete a distinct pattern of proteins (including distinct abundance and diversity).

All these results show that *S.* Typhi Δ*zzz* mutants can secrete a distinct pattern of proteins, including functional periplasmic proteins such as HlyE and β-lactamase.

### Characterization of *S.* Typhi Δ*zzz* Regarding the Envelope Integrity

**FIGURE 3:**
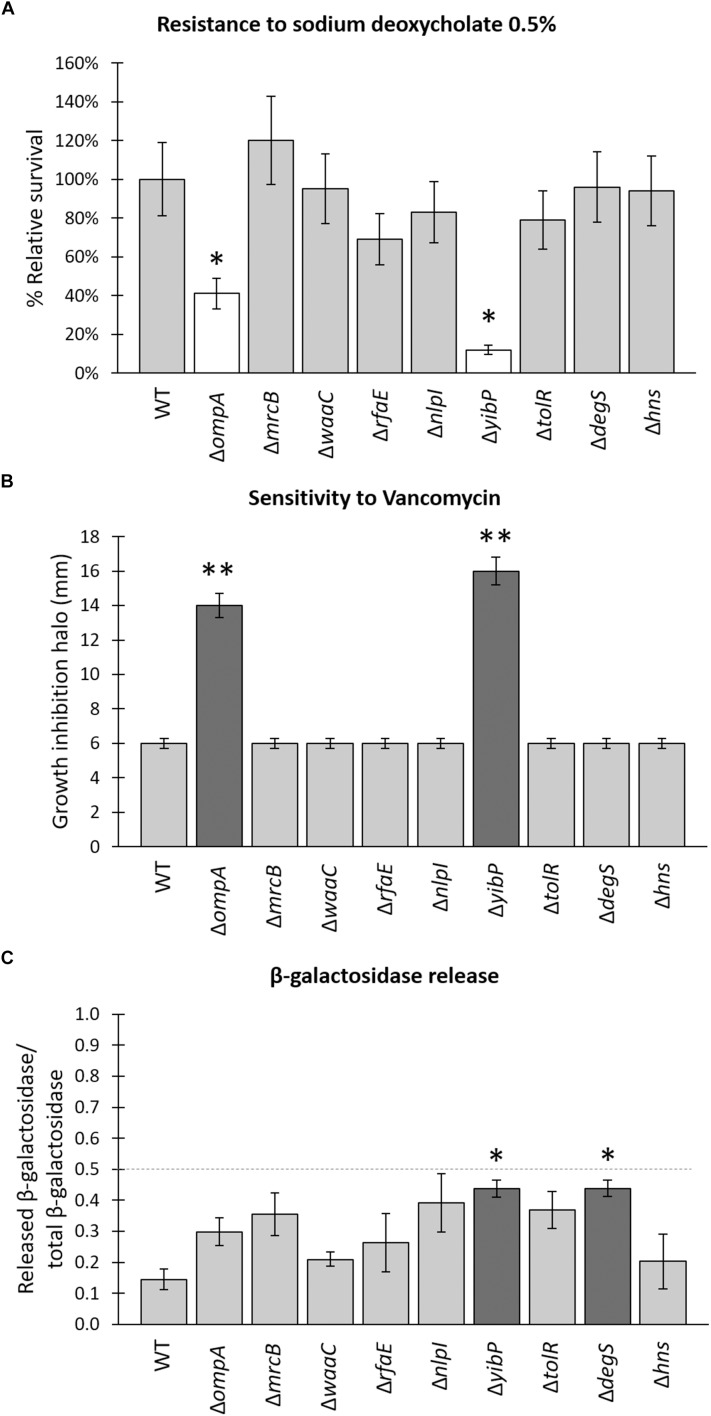
Resistance to deoxycholic acid and vancomycin, and β-galactosidase release of *S.* Typhi WT and Δ*zzz* mutants. **(A)** Resistance to sodium deoxycholate 0.5%. Bacteria were grown in LB with shaking at 37°C to OD_600_ = 0.5, prior to being treated during 2 h with sodium deoxycholate 0.5% or PBS (control) at 37°C. Percentage of survival was calculated as (cfu treated with sodium deoxycholate/cfu with PBS) × 100, and the relative values with respect to the WT were shown. **(B)** Kirby–Bauer assay to determine the sensitivity to vancomycin (30 μg in the paper disk) of *S.* Typhi WT and *S.* Typhi Δ*zzz* mutants. The *Y*-axis shows the growth inhibition halo (diameter) measured in mm. 6 mm corresponds to the technique detection limit determined by the paper disk diameter. **(C)** Determination of β-galactosidase release. *S.* Typhi WT and *S.* Typhi Δ*zzz* mutants expressing β-galactosidase (from a religated version of pCR TOPO TA [Lac^+^] plasmid) were grown in LB to stationary phase prior to obtaining the pellet and the supernatant fraction by centrifugation. β-galactosidase of each fraction was determined and used to calculate the enzyme release as stated in Materials and Methods. In all cases, dark gray and white bars show positive and negative (^∗^*p* < 0.05, ^∗∗^*p* < 0.01) statistically significant differences, compared with *S.* Typhi WT (*n* = 4 for each case, Kruskal–Wallis with Dunn’s test as *post hoc* analysis).

It has been reported that *S.* Typhimurium mutants lacking OmpA, LppAB, Pal, TolA, or TolB exhibit an increased production of OMVs (Deatherage et al., 2009). Nevertheless, those mutants also present severe problems regarding membrane integrity, leading to high susceptibility to bile acids (e.g., sodium deoxycholate). This phenomenon was also observed in other Gram-negative bacteria (McBroom et al., 2006). In this sense, it has been proposed that mutants producing more subtle changes in envelope crosslinking can reveal more physiological conditions involved in OMVs biogenesis (i.e., “subtle mutants”). To check the envelope integrity and to determine whether the *S.* Typhi Δ*zzz* mutants are more susceptible to sodium deoxycholate, bacteria grown to approximately OD_600_ = 0.5 were challenged with 0.5% deoxycholate or PBS (control) for 2 h. As shown in Figure 3A, only *S.* Typhi Δ*ompA* and *S.* Typhi Δ*yibP* exhibited a statistically significant increased susceptibility to sodium deoxycholate. The same experiment performed with bacteria grown to approximately OD_600_ = 1.1, and including complementation of the mutations, gave similar results (Supplementary Figure 3). To further estimate the envelope integrity, we determined the relative susceptibility to vancomycin by a Kirby–Bauer assay. Gram-negative bacteria are intrinsically resistant to vancomycin because of the limit of diffusible molecules through the bacterial envelope (Pimenta et al., 1999). Thus, an increased susceptibility to vancomycin could be interpreted as an increased permeability. We found that almost all the *S.* Typhi mutants exhibited complete resistance to vancomycin, except for *S.* Typhi Δ*ompA* and *S.* Typhi Δ*yibP* (Figure 3B). These same mutants showed increased sensitivity to sodium deoxycholate, strongly suggesting that the envelope may be compromised in these cases. The remaining mutants were undistinguishable from the WT strains (i.e., “subtle mutants”). Remarkably, when *S.* Typhimurium and the corresponding *S.* Typhimurium Δ*zzz* mutants were evaluated, a different pattern of sensitivity was observed (Supplementary Figure 4), reinforcing the fact that data obtained from *S.* Typhimurium cannot be freely extrapolated to *S.* Typhi without an experimental approach.

To better characterize the *S.* Typhi Δ*zzz* mutants regarding their envelope integrity, we assessed the release of cytoplasmic proteins. β-galactosidase release has been used as an indicator of cell disruption in many cell types, including *S. enterica* (Kong et al., 2008). Thus, we transformed the pCR TOPO TA^®^ religated, multicopy plasmid (*lacZ*^+^) into *S.* Typhi WT and *S.* Typhi Δ*zzz* mutants, to generate the corresponding Lac^+^ version of these strains. Since *S.* Typhi lacks *lacI* (Parkhill et al., 2001; Valenzuela et al., 2014), the Lac^+^ phenotype is constitutive and presented no statistically differences among *S.* Typhi Δ*zzz* mutants (Supplementary Figure 5). As shown in Figure 3C, the ratio of β-galactosidase activity in the supernatant versus total β-galactosidase activity indicated leakage of cytoplasmic enzymes. Although most *S.* Typhi Δ*zzz* mutants seem to release more β-galactosidase than the WT, only *S.* Typhi Δ*yibP* and Δ*degS* exhibited statistically significant differences. Since it has been considered that a ratio superior to 0.5 could correspond to cell lysis (Kong et al., 2008), we speculate that *S.* Typhi Δ*yibP* and Δ*degS* present some leakage of cytoplasmic enzymes, where bacterial lysis is not the most important factor, and such leakage is less pronounced in the other *S.* Typhi Δ*zzz* mutants. Altogether, these results show that most *S.* Typhi Δ*zzz* can be considered as “subtle mutants” since they exhibit a good envelope integrity and a relatively low leakage of cytoplasmic proteins, even though the reporter gene (*lacZ*) is in a high gene dosage in this case.

### Characterization of Outer Membrane Vesicles Produced by *S.* Typhi Δ*zzz* Mutants

**FIGURE 4:**
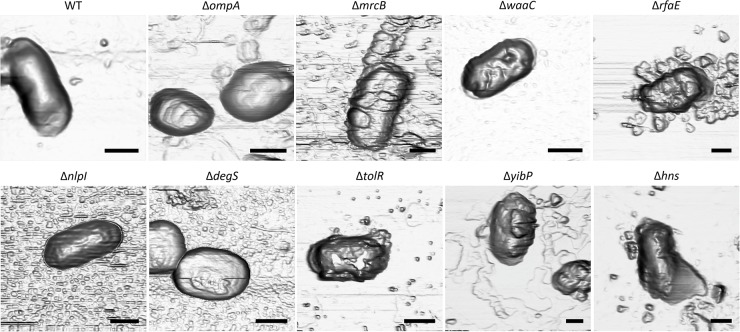
Atomic force microscopy (AFM) of *S.* Typhi WT and *S.* Typhi Δ*zzz* mutants. AFM images of bacterial surface. Bacteria were cultured in LB at 37°C with shaking to stationary phase. The black bar corresponds to 1 μm. In each case, a representative experiment is shown (*n* = at least 3).

**FIGURE 5:**
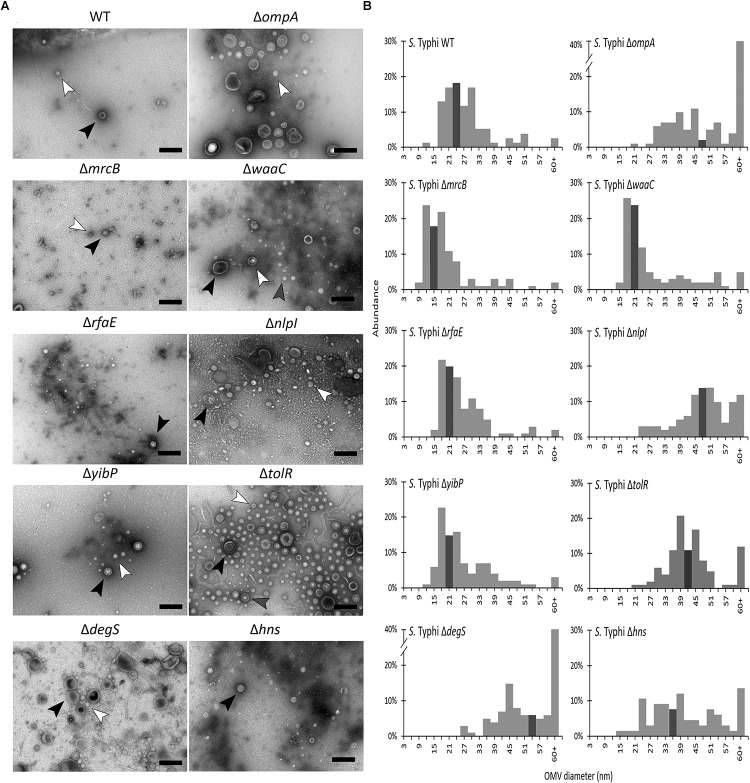
Deletion of *zzz* genes impacts on the distribution of Outer Membrane Vesicle (OMV) size. **(A)** OMV extracts were observed by transmission electronic microscopy (TEM). The black bar corresponds to 200 nm. In all cases the horizontal field width and the magnification corresponded to 1.5 μm and 60000×, respectively. The black, gray, or white arrowhead show OMVs with different sizes. In each case, a representative experiment is shown (*n* = at least 3). **(B)** Size distribution of OMVs produced either by *S.* Typhi WT or *S.* Typhi Δ*zzz* mutants. OMV size was obtained from TEMs (see Materials and Methods, Supplementary Table 2 and Supplementary Figure 7 for details). Dark bar contains the median for each case.

At this point, we have evidence arguing for the role of *zzz* genes in OMV biogenesis: (1) all the *S.* Typhi Δ*zzz* mutants export increased amounts of periplasmic proteins (i.e., HlyE and β-lactamase) (Figure 1, 2), where *S.* Typhi HlyE is known to be secreted via OMVs (Wai et al., 2003); (2) *S.* Typhi Δ*zzz* mutants have a distinct protein secretion into the supernatant fraction (Figure 3); (3) all *S.* Typhi Δ*zzz* mutants, except for *S.* Typhi Δ*ompA* and *S.* Typhi Δ*yibP*, exhibited a good envelope integrity, compared with the WT (Figure 3); and (4) all *zzz* genes, except *hns*, encode functions clearly related to the bacterial envelope (Table 1). In this context, we examined bacterial surface by AFM to determine whether Δ*zzz* deletions produce envelope changes that could be associated to OMV biogenesis. AFM allows obtaining high-resolution images of bacterial samples, measuring structures in scales from molecules to cells (Liu and Wang, 2010). As shown in Figure 4, *S.* Typhi WT exhibited a smooth surface. By contrast, we found that, in all the cases, *S.* Typhi Δ*zzz* mutants presented some changes in bacterial surface, such as waves, protrusions, creases or structural modifications that could correspond to envelope evaginations, consistent with a potential hypervesiculator phenotype (see Supplementary Figure 6 to complement the analysis). In the case of *S.* Typhi Δ*yibP*, we found two cell configurations: filament-like (Supplementary Figure 7) and isolated (Figure 4). On the other hand, several structures, consistent with released OMVs, can be observed in *S.* Typhi Δ*zzz* mutants. Similar images obtained by AFM have been previously interpreted as OMV release (Wai et al., 2003; Chatterjee and Chaudhuri, 2011; Thoma et al., 2018). In this sense, *ompA* has been previously reported as involved OMV biogenesis in *S.* Typhimurium (Deatherage et al., 2009), and *zzz* genes are encoding different functions related to the bacterial envelope. For these reasons, we hypothesized that *S.* Typhi Δ*zzz* are producing distinct OMVs, exhibiting different structural features, such as the size distribution, and potentially distinct OMV cargo. Thus, we isolated OMVs from *S.* Typhi WT and Δ*zzz* mutants, prior to observing them with transmission electronic microscopy. As shown in Figure 5A, *S.* Typhi Δzzz mutants produced morphologically distinct OMVs, showing different mean sizes and more variability compared with OMVs produced by the *S.* Typhi WT. Δ*zzz* deletions affected OMV size and/or OMV size distribution, where *S.* Typhi Δ*ompA*, Δ*tolR*, Δ*degS*, and Δ*hns* tended to produce bigger OMVs compared with the WT, *S.* Typhi Δ*mrcB* produced smaller OMVs, and *S.* Typhi Δ*waaC* was prone to produce both smaller and bigger OMVs. Nevertheless, all mutants seemed to produce more variable OMVs regarding their size, when compared with WT OMVs (Figure 5B and Supplementary Figure 8, see Supplementary Table 2 for statistical analysis). In addition, we observed that the increased β-lactamase release found in *S.* Typhi Δ*att*_BG1_::pNFB9 Δzzz double mutants (Figure 2) depends, at least in part, on the secretion of OMVs (Supplementary Figure 9). On the other hand, we were able to observe the direct release of OMVs from *S.* Typhi Δ*tolR* and Δ*degS*, where differences in OMV size were also evident (Supplementary Figure 10).

**FIGURE 6:**
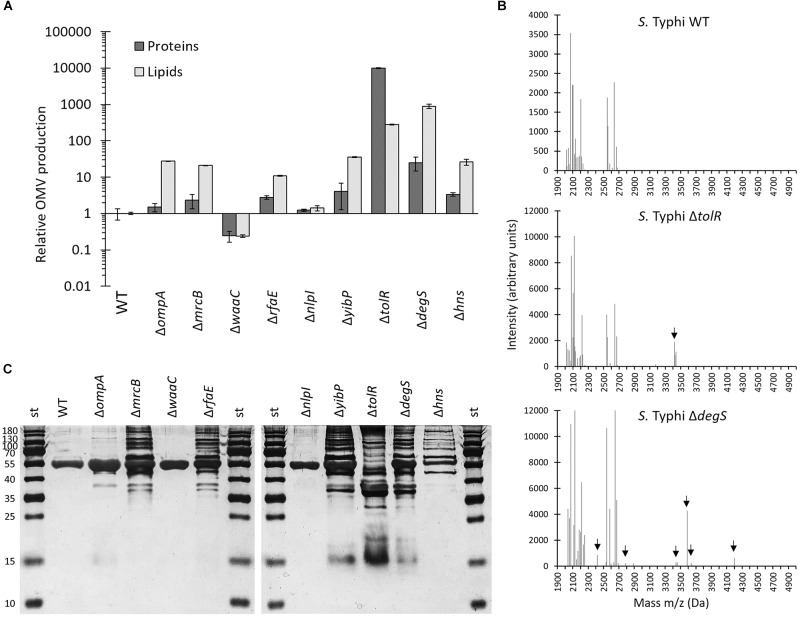
**(A)** OMV production of *S.* Typhi WT and *S.* Typhi Δ*zzz* mutants. Bacteria were grown in LB to stationary phase, and OMVs were extracted. Relative fold of OMV production was obtained by quantitating both the protein and lipid content (McBroom et al., 2006; Deatherage et al., 2009; Schwechheimer et al., 2015), normalizing to CFU/ml, and dividing by (CFU/ml)-normalized OMV production obtained with the *S.* Typhi WT. **(B)** MALDI-TOF mass spectrometry of OMV extracts. Arrows indicate differences with *S.* Typhi WT (*n* = 2, representative experiment). **(C)** SDS–PAGE of OMV extracts obtained from *S.* Typhi WT and *S.* Typhi Δ*zzz* mutants. Proteins were visualized by silver staining.

To determine the OMV yield of *S.* Typhi Δ*zzz* mutants, we performed two independent strategies. To this end, we cultured both the *S.* Typhi WT and the *S.* Typhi Δ*zzz* in LB to stationary phase (OD_600_ = 1.1), and OMVs were extracted. Then, we determined the protein content of OMVs as an indicator of abundance, as previously described, normalizing with CFU/ml (Deatherage et al., 2009). As shown in Figure 6A and Supplementary Figure 11, *S.* Typhi Δ*tolR* and *S.* Typhi Δ*degS* clearly presented more proteins in the OMV fraction, whereas *S.* Typhi Δ*ompA*, Δ*mrcB*, Δ*rfaE*, Δ*yibP*, and Δ*hns* exhibited a slight but consistent increase in the OMV protein content. On the other hand, *S.* Typhi Δ*nlpI* exhibited similar results compared with *S.* Typhi WT and, strikingly, *S.* Typhi Δ*waaC* seems to present less proteins in the OMV fraction. Considering that the heterogeneity in OMV size and the leakage of cytoplasmic proteins, such as in the Δ*degS* mutant, could affect the protein content in the OMV fraction, we also performed a direct quantification of lipids in OMV preparation for vesicle quantification, as previously described. As well as for protein content, we also normalized our results with CFU/ml in each case (McBroom et al., 2006). As shown in Figure 6A and Supplementary Figure 11, the lipid content determination corroborated the results obtained with the protein content determination. An increase in both protein and lipid content in the OMV fraction have been previously interpreted as an increased OMV yield, and vice versa (McBroom et al., 2006; Deatherage et al., 2009; Schwechheimer et al., 2015). Interestingly, *S.* Typhi Δ*waaC* released more HlyE and β-lactamase to the supernatant, compared with the WT, when 5 μl of a stationary culture were plated on agar plates (Figure 1, 2), but produced less OMVs per CFU. This result could be explained by the fact that *S.* Typhi Δ*waaC* exhibited 10-fold more CFU/ml in a stationary culture, compared with the WT (Supplementary Figure 12).

Altogether, these results indicate that *zzz* genes participate in the OMV biogenesis, affecting OMV production. This assertion is supported by the AFM (Figure 4), where Δ*zzz* mutants seem to show structures potentially corresponding to a hypervesiculating phenotype.

As stated above, protein cargo selection is a process that should occur during OMV biogenesis (Schwechheimer and Kuehn, 2015). Since *zzz* genes participate in the OMV biogenesis, we expect that cargo in OMVs derived from *S.* Typhi Δ*zzz* mutants should be affected. To test this hypothesis, we performed MALDI-TOF mass spectrometry of OMVs extracts. We found that most mutants produced OMVs presenting MALDI-TOF profiles that were very similar to that obtained with the WT OMVs, except for *S.* Typhi Δ*nlpI*-derived OMVs, which exhibited slight differences (Supplementary Figure 13), and OMVs obtained from *S.* Typhi Δ*tolR* and Δ*degS* (Figure 6B), which showed more evident differences. These results indicate that, at least for *tolR* and *degS, zzz* genes participate in processes that modulate OMV cargo. To observe potential differences in protein cargo in an independent way, we visualized OMV proteins with SDS–PAGE. As shown in Figure 6C, OMVs derived from *S.* Typhi WT exhibited few observable proteins, with a high abundance of a protein of approximately 55 kDa (plausibly corresponding to flagellin). This pattern is similar to OMV extracts derived from other WT *S. enterica* serovars (Liu et al., 2016a, 2017). We observed that protein cargo of OMVs derived from *S.* Typhi Δ*zzz* mutants was different to that observed for the WT OMVs, corroborating the role of these genes in OMV cargo selection. Interestingly, when OMVs obtained from *S.* Typhi Δ*mrcB* were analyzed, we also observed differences in comparison with the WT OMVs. Although OMVs derived from *S.* Typhi WT and *S.* Typhi Δ*mrcB* presented a similar MALDI-TOF pattern (Supplementary Figure 13), it is possible that more subtle differences cannot be detected by the methods performed in this work. Accordingly, albeit many bands are shared between WT OMVs and Δ*mrcB* OMVs (Figure 6C), their abundance differs. The same could be extrapolated to the other Δ*zzz* mutants. In this sense, a more profound study must be performed to determine how exactly *zzz* are affecting OMV cargo.

According to these results, we conclude that *zzz* genes participate in OMV biogenesis in *S.* Typhi, affecting OMV size distribution, OMV production, and, at least for *tolR* and *degS*, we conclude that *zzz* are involved in OMV cargo selection.

## Discussion

In this work, we aimed to identify genes involved in OMV biogenesis in *S.* Typhi. To this purpose, we performed a transposon-based screening that allowed identifying 9 genes that participate in OMV biogenesis. The fact that many of the identified genes were found more than once in the screening (Table 1), and considering that the screening allowed us identifying *ompA* and *hns*, two genes already reported as involved in HlyE secretion and in repression of *hlyE* expression, respectively (Oscarsson et al., 2002; Fuentes et al., 2008), indicates that the screening depth was suitable, and the whole genome was explored. Although transposon mutagenesis was previously described as a useful tool to identify genes potentially involved in HlyE export in an HlyE-overproducing *E. coli* strain (Wyborn et al., 2004a), to our knowledge, this is the first time that the hemolytic phenotype is used to identify genes involved in OMV biogenesis.

Our analysis showed that *ompA, tolR* and *nlpI* (envelope stability), *rfaE* and *waaC* (LPS synthesis), *yipP, mrcB* (synthesis and remodeling of peptidoglycan), *degS* (stress sensor serine endopeptidase) and *hns* (global transcriptional regulator) were involved in HlyE-dependent hemolysis in *S.* Typhi. We showed that this phenotype is mainly due to an increased export of HlyE rather than to an increased *hlyE* expression, except for the *S.* Typhi Δ*hns* mutant. The role of HNS in *hlyE* repression was already reported for *S.* Typhi (Oscarsson et al., 2002) and *E. coli* (Wyborn et al., 2004b).

Outer membrane vesicle biogenesis is poorly understood (Deatherage et al., 2009) and unknown in *S.* Typhi. Nevertheless, there is evidence showing that this process mainly relies on the dissociation of the outer membrane in specific zones lacking proper attachments to underlying structures (e.g., peptidoglycan). Studies performed in *E. coli* revealed that envelope stability comes from three main crosslinks: OmpA, an outer membrane protein linked to peptidoglycan (Park et al., 2012), LppAB, highly abundant outer membrane lipoproteins covalently crosslinked to peptidoglycan (Mizushima, 1984) and the Tol-Pal complex, a cell division component that contributes to envelope stability also by interacting with peptidoglycan (Cascales et al., 2002; Gerding et al., 2007; Yeh et al., 2010). When these crosslinks decrease, production of OMVs increases, and vice versa. Accordingly, *S.* Typhimurium mutants lacking OmpA, LppAB, Pal, TolA, or TolB exhibit an increased production of OMVs (Deatherage et al., 2009). Nevertheless, these mutants also present severe problems regarding membrane integrity, leading to high susceptibility to bile acids and cellular leakage (Sonntag et al., 1978; McBroom et al., 2006; Deatherage et al., 2009). In this work, we found that *ompA* is involved in OMV biogenesis in *S.* Typhi, reinforcing the fact that the screening was suitable to identify OMV-related genes in this serovar. Interestingly, *S.* Typhimurium Δ*ompA* (Deatherage et al., 2009) apparently produces smaller OMVs than *S.* Typhi Δ*ompA* (Figure 5), remarking potential differences regarding OMV biogenesis between these two serovars. On the other hand, we were unable to find mutants in the Braun (murein) lipoprotein gene *lpp*. The genome of *S.* Typhimurium presents two copies of *lpp* (i.e., *lppA* or *lpp1*, and *lppB* or *lpp2*) (Sha et al., 2004). Similarly, *S.* Typhi genome also harbors two copies of *lpp* (Parkhill et al., 2001; Valenzuela et al., 2014). This fact might remark a limitation of the screening, indicating that genes encoding redundant functions related to OMV biogenesis cannot be identified with the proposed strategy. However, this same finding remarks an advantage of the screening, showing that the identified genes encode specific functions that are not redundant regarding OMV biogenesis. In other words, each *zzz* gene contributes to OMV biogenesis in *S.* Typhi by a specific, unique mechanism, underlying the contribution of this work. This point is supported by the fact that *S.* Typhi Δ*zzz* mutants produce distinct OMVs regarding size distribution, abundance and protein cargo.

TolR, part of the Tol-Pal system (TolA, TolB, TolQ, and TolR), is an inner membrane protein related to the peptidoglycan-binding stator from the flagellum in *E. coli*. The Tol-Pal systems contribute to maintaining integrity of the outer membrane (Deatherage et al., 2009). Although TolR is conserved among Gram-negative bacteria, some important structural differences among related bacteria have been reported (Wojdyla et al., 2015). TolA, TolB, and Pal have been shown to affect OMV biogenesis in *S.* Typhimurium (Deatherage et al., 2009), albeit we were unable to find the corresponding encoding genes as implicated in *S.* Typhi OMVs. As stated, it is possible that differences in the Tol-Pal system between these two serovars could explain this result. For instance, TolA determines an increase resistance to bile in *S.* Typhi, compared with *S.* Typhimurium (Lahiri et al., 2011), and *tolR* encodes functions involved in motility only in *S.* Typhimurium, and *E. coli* (Santos et al., 2014), but not in *S.* Typhi (Supplementary Figure 14). Interestingly, in this work we showed that *S.* Typhi Δ*tolR* produce high amount of OMVs, without an important compromise of the cell envelope, presenting the same resistance to deoxycholate than the WT (i.e., a “subtle mutant). By contrast, *S.* Typhimurium Δ*pal*, Δ*tolA*, and Δ*tolB* mutants exhibit a highly compromised envelope stability (Deatherage et al., 2009), suggesting that deletions in different components of the Tol-Pal systems are not fully equivalent. In support of this point, we found that *S.* Typhimurium Δ*tolR* produces only 2-fold more OMVs compared with *S.* Typhimurium WT, remarking another difference between *S.* Typhi and *S.* Typhimurium (Supplementary Figure 15).

NlpI, a lipoprotein that participates in the balance of peptidoglycan breakdown and synthesis, partially determines (around 40%) the formation of Lpp-peptidoglycan crosslinks (Schwechheimer et al., 2015). In this sense, *E. coli* Δ*nlpI* exhibits an increased OMVs production compared to the otherwise isogenic parental strain, without evident leakage of cytoplasmic proteins (i.e., *E. coli* Δ*nlpI* is a subtle mutant) (McBroom et al., 2006; Schwechheimer et al., 2015). According to our results, *S.* Typhi Δ*nlpI* (as well as Δ*mrcB*, Δ*waaC*, Δ*rfaE*, Δ*yibP*, Δ*tolR*, Δ*degS*, and Δ*hns*) can be also considered subtle mutants since OMV production is independent of envelope instability in this serovar (Figure 3). In the best scenario, it might be possible to modify OMV production without compromising bacterial global envelope integrity, providing a potential improved genetic background to perform further OMV proteomic analyses (with few contamination of cytoplasmic proteins) or for biotechnological purposes (Bonnington and Kuehn, 2014; Schwechheimer and Kuehn, 2015). On the other hand, although *nlpI* has been associated to hypervesiculation in other Gram-negative bacteria (e.g., *Actinobacillus pleuropneumoniae*) (Antenucci et al., 2017), its role in *S. enterica* is less known, where it has been linked to biofilm formation and acclimatization in *S.* Typhimurium (Rouf et al., 2011a,b).

It has been stated that LPS remodeling triggers formation of OMVs in *S. enterica* (Bonnington and Kuehn, 2016; Elhenawy et al., 2016). Nevertheless, although several genes are involved in LPS synthesis, we only found, *waaC* and *rfaE* in the screening. The *waaC* gene encodes a heptosyltransferase, an enzyme that catalyzes the transfer of the first heptose to the lipid A in the core region of the LPS, as described for *E. coli* (Durka et al., 2012). On the other hand, *rfaE* encodes an enzyme harboring two domains that catalyze the synthesis of the LPS core precursor ADP-L-glycero-D-manno-heptose, as reported for *E. coli* (Valvano et al., 2000). Although these two enzymes (WaaC and RfaE) work together in the same LPS synthetic pathway, they are not redundant. In fact, although both *waaC* and *rfaE* affect OMV biogenesis in *S.* Typhi, *S.* Typhi Δ*waaC* produced less OMVs than the WT, whereas *S.* Typhi Δ*rfaE* can be considered as a hypervesiculating strain. In support of this, a deletion in *waaC* in *S.* Typhimurium inhibits vesiculation (Liu et al., 2016b). Nevertheless, *waaC* and *rfaE* seem to be only partially equivalent between *S.* Typhimurium and *S.* Typhi, since their respective deletions apparently affect in different ways the motility of these two serovars (Supplementary Figure 14). As stated, changes in LPS composition can affect OMV production, presumably by generating local nanoterritories in the outer membrane. These nanoterritories could exhibit differential curvature, fluidity, and/or charge, making the membrane more or less prone to be discharged, depending on the modification (Haurat et al., 2011). Furthermore, only a subset of genes involved in LPS have shown to participate in OMV biogenesis (Haurat et al., 2011; Elhenawy et al., 2016), assertion that is supported by the results of our screening.

The *mrcB* gene encodes the PBP-1b (penicillin binding protein). In *E. coli*, PBP-1b corresponds to a bifunctional enzyme (transglycosidase and transpeptidase activity) that participates in synthesis of peptidoglycan (Sauvage et al., 2008; Kumar et al., 2012). Since changes in peptidoglycan dynamics (i.e., decrease in covalent cross-links between outer membrane and bacterial wall) can induce OMV production, we speculate that *mrcB* encodes functions that could also contribute to this process in *S.* Typhi. In support of this, it has been reported that PBP-1b provides an important physical link between the inner and outer membranes at the division site in *E. coli* (Markovski et al., 2016). Thus, the lack of *mrcB* could impact on envelope stability, affecting vesiculation. On the other hand, *yibP* (also known as *envC*) encodes an enzyme showing a murein hydrolytic activity related to peptidoglycan hydrolysis associated with cell division. YibP interacts with FtsX and FtsZ in bacterial septa (Yang et al., 2011), as well as with amidases that cleave the septal murein and promotes daughter cell separation (Bernhardt and de Boer, 2004). The lack of *yibP* results in defects of septation, producing filament-like cells in *E. coli* (Hara et al., 2002), a phenotype that we also found in *S.* Typhi Δ*yibP* (Supplementary Figure 7). Interestingly, it has been stated that the interactions of the outer membrane, the peptidoglycan and the inner membrane at division septa contribute to the envelope integrity and modulate localized release of OMVs, affecting both the size distribution and the protein content of the OMVs in *S.* Typhimurium (Deatherage et al., 2009). In this sense, our results with the *S.* Typhi Δ*yibP* could be indicating that specific or particular functions related to septa formation are also involved in OMV biogenesis. To our knowledge, neither *mrcB* nor *yibP* were previously described to be associated to OMV biogenesis.

In *S.* Typhimurium and in *E. coli, degS* encodes an inner-membrane-anchored periplasmic endopeptidase belonging to the HtrA family (Rhodius et al., 2006; Clausen et al., 2011). In presence of unfolded proteins (e.g., outer membrane proteins), DegS cleaves the cytoplasmic moiety of RseA (also known as anti-σ^E^), releasing σ^E^ and activating the transcription of genes involved in repair and/or elimination of misfolded periplasmic proteins (Missiakas et al., 1997). DegS could participate in OMV biogenesis by both a direct and an indirect mechanism: First, in absence of DegS, misfolded proteins could accumulate in the periplasmic space. This accumulation in distinct nanoterritories may cause displacement or depletion of local crosslinks, destabilizing the bacterial envelope and inducing vesiculation (Bonnington and Kuehn, 2014; Schwechheimer and Kuehn, 2015). And second, the lack of DegS impairs the activation (release) of σ^E^. In this sense, the impairment of the σ^E^ pathway positively affects vesiculation, as shown for *E. coli* (Button et al., 2007; McBroom and Kuehn, 2007).

The *hns* gene encodes the global regulator H-NS reported to silence genes acquired by horizontal transfer by counteracting transcription of AT-rich promoter-like regions (Singh et al., 2014). Accordingly, H-NS acts as a repressor of *hlyE*, belonging to the SPI-18, a genomic locus presumably acquired by horizontal transfer in *S.* Typhi (Oscarsson et al., 2002; Fuentes et al., 2008). Interestingly, we found that *hns* is also involved in OMV biogenesis in this serovar. Previously, it has been reported that *E. coli* Δ*hns* exhibits an increased OMV yield, supporting the role of *hns* in OMV biogenesis in *Enterobacteriaceae* (Horstman and Kuehn, 2002; Sanchez-Torres et al., 2010). Although the mechanism by which H-NS participates in OMV biogenesis is unknown, it is possible that this regulator is involved in the expression of other genetic determinants of vesiculation, as previously proposed (Horstman and Kuehn, 2002).

Regarding the regulation of vesiculation, it has been shown that HlyE secretion, which occurs via OMVs in *S.* Typhi (Wai et al., 2003), is indirectly promoted by the downregulation of OmpA in presence of epinephrine or norepinephrine, hormones that are abundant in the gut or in macrophages exposed to LPS (Furness, 2000; Flierl et al., 2007, 2009; Karavolos et al., 2011). Thus, the identification of new genetic determinants of OMV biogenesis in *S.* Typhi could contribute to a better understanding of how vesiculation is regulated during the normal infective cycle. In other words, the study of *zzz* regulation, in the OMV context, could provide clues about the exact role of HlyE in the normal infective cycle. Furthermore, even though there are no good animal models for *S.* Typhi, these mutants could be also used in future tissue culture and macrophage studies.

Since proteins are the most significant determinants of OMV functions (Lee et al., 2016), identification of OMV-associated proteins is crucial. In this work, we showed that that some Δ*zzz* deletions affected protein content of *S.* Typhi-derived OMVs. It has been stated that genes involved in OMVs biogenesis are also involved in inclusion/exclusion of proteins in OMVs, supporting the assertion that OMV cargo selection is determined during OMV biogenesis (Schwechheimer and Kuehn, 2015). For instance, *Pseudomonas aeruginosa* lacking OprF, a homologous of OmpA, exhibits an increased production of OMVs. Furthermore, OprF impacts on OMV cargo, modulating both abundance and diversity of OMV-associated proteins (Wessel et al., 2013). On the other hand, DegP, a chaperone participating in protein-folding quality control of OM proteins (Clausen et al., 2011), contributes to both OMV protein cargo selection and to OMVs functions in *Vibrio cholerae* (Altindis et al., 2014). OMVs from *V. cholerae* Δ*degP* lack proteins involved in biofilm formation; thereby, these mutants are impaired in biofilm biogenesis (Wessel et al., 2013). Remarkably, DegP is important in OMV biogenesis in *E. coli* (McBroom et al., 2006). Furthermore, deletion of genes contributing to LPS composition affects OMV production in *S.* Typhimurium (Elhenawy et al., 2016), as well as OMV production, cargo selection, and even OMV size in *P. aeruginosa* (Murphy et al., 2014). All this evidence argues that genes implicated in different stages of OMV biogenesis are also involved in OMV cargo selection, potentially determining OMV functions and other properties such as OMV size (Yoon, 2016).

Since OMVs are vehicles for bacterial proteins, their study will allow getting a better insight of the communication occurring between bacteria–host cells, potentially allowing the identification of new proteins involved in virulence. On the other hand, since OMVs can also be used to deliver heterologous antigens in vaccine design (van der Pol et al., 2015), a better understanding of OMV biogenesis in *S. enterica*, a species used to develop recombinant attenuated vaccines (Curtiss et al., 2010), will allow engineering improved OMVs. Thus, the study of OMVs produced by *S.* Typhi Δ*zzz* mutants could potentially contribute to better comprehend their role in the infection cycle, but also for the development of improved biotechnological tools.

## Dedication

This manuscript is dedicated to Prof. Guido C. Mora, for a whole life devoted to science and for his contribution to the microbiology in Chile and the world.

## Author Contributions

JN performed the experiments of the Figure 1–3 and Supplementary Figures 1–7, 14. AS peformed the experiments of the Figure 4–6A,C and Supplementary Figures 8–12, 15. CO critically read the manuscript and co-directed the project. DO contributed with the data for Figure 4 and Supplementary Figure 6. BB contributed with the data for Figure 6B and Supplementary Figure 13. FG and IC critically read the manuscript and discussed the results. JF designed and discussed all the experiments, designed all the figures, wrote the manuscript, and directed the project.

## Conflict of Interest Statement

The authors declare that the research was conducted in the absence of any commercial or financial relationships that could be construed as a potential conflict of interest.
