# Supplementary material for: Identification of Genes Involved in Biogenesis of Outer Membrane Vesicles (OMVs) in Salmonella enterica Serovar Typhi
Source: Front Microbiol. 2019 Feb 4;10:104. doi: 10.3389/fmicb.2019.00104 (PMC6369716; doi:10.3389/fmicb.2019.00104)
Supplement: Supplementary file 1 [file Presentation_1.PPTX]

## Slide 1
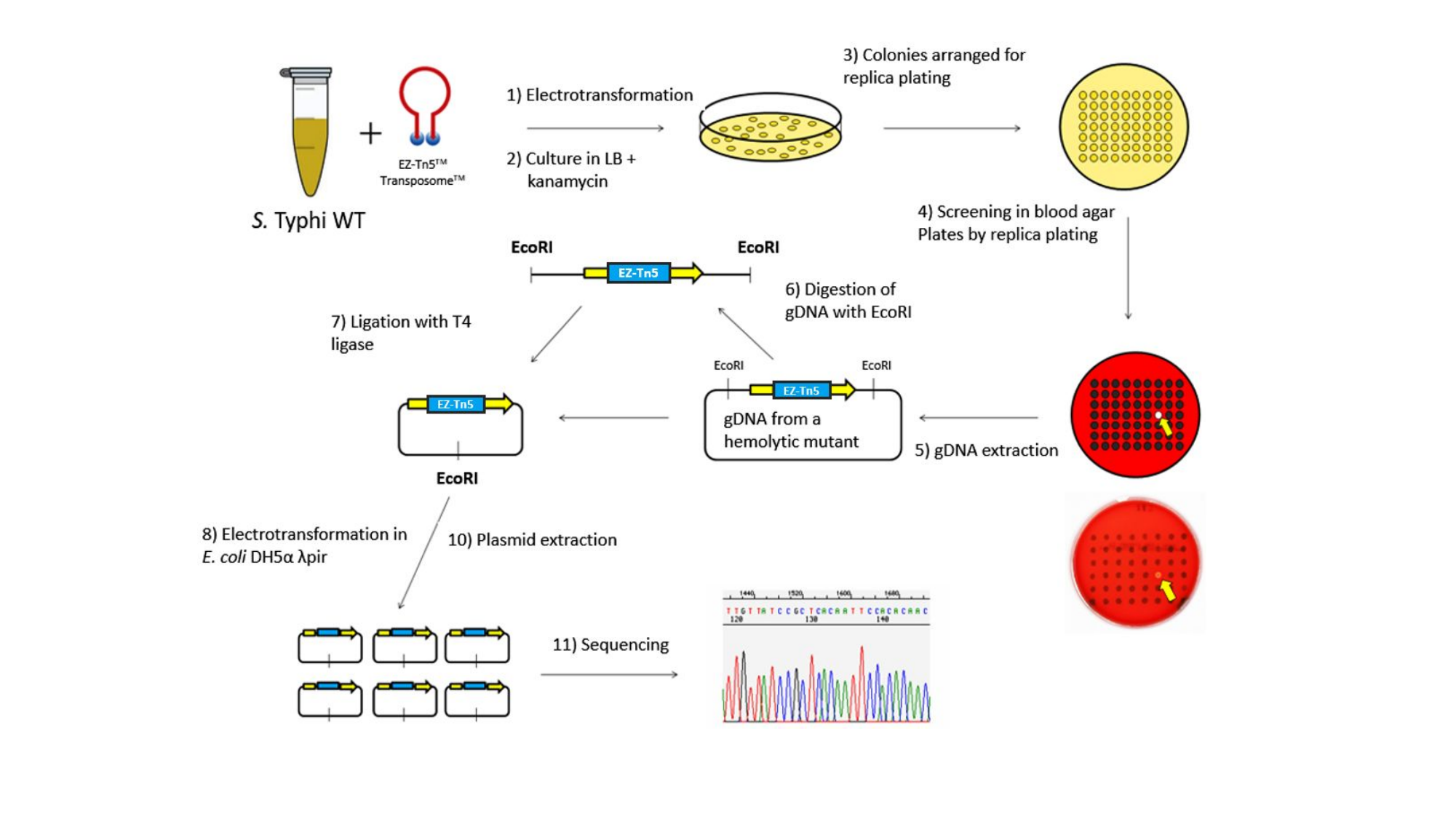

## Slide 2
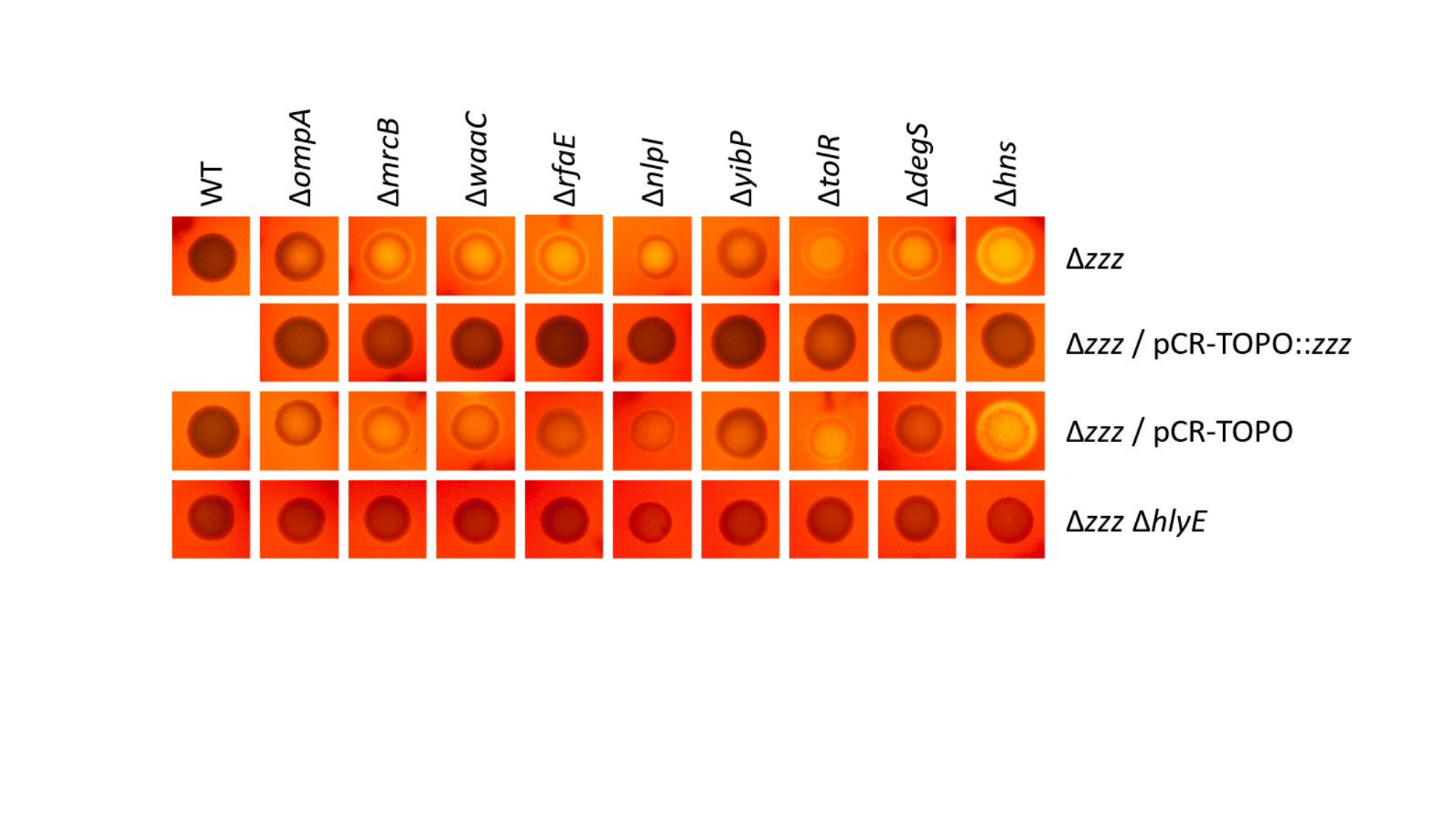

## Slide 3
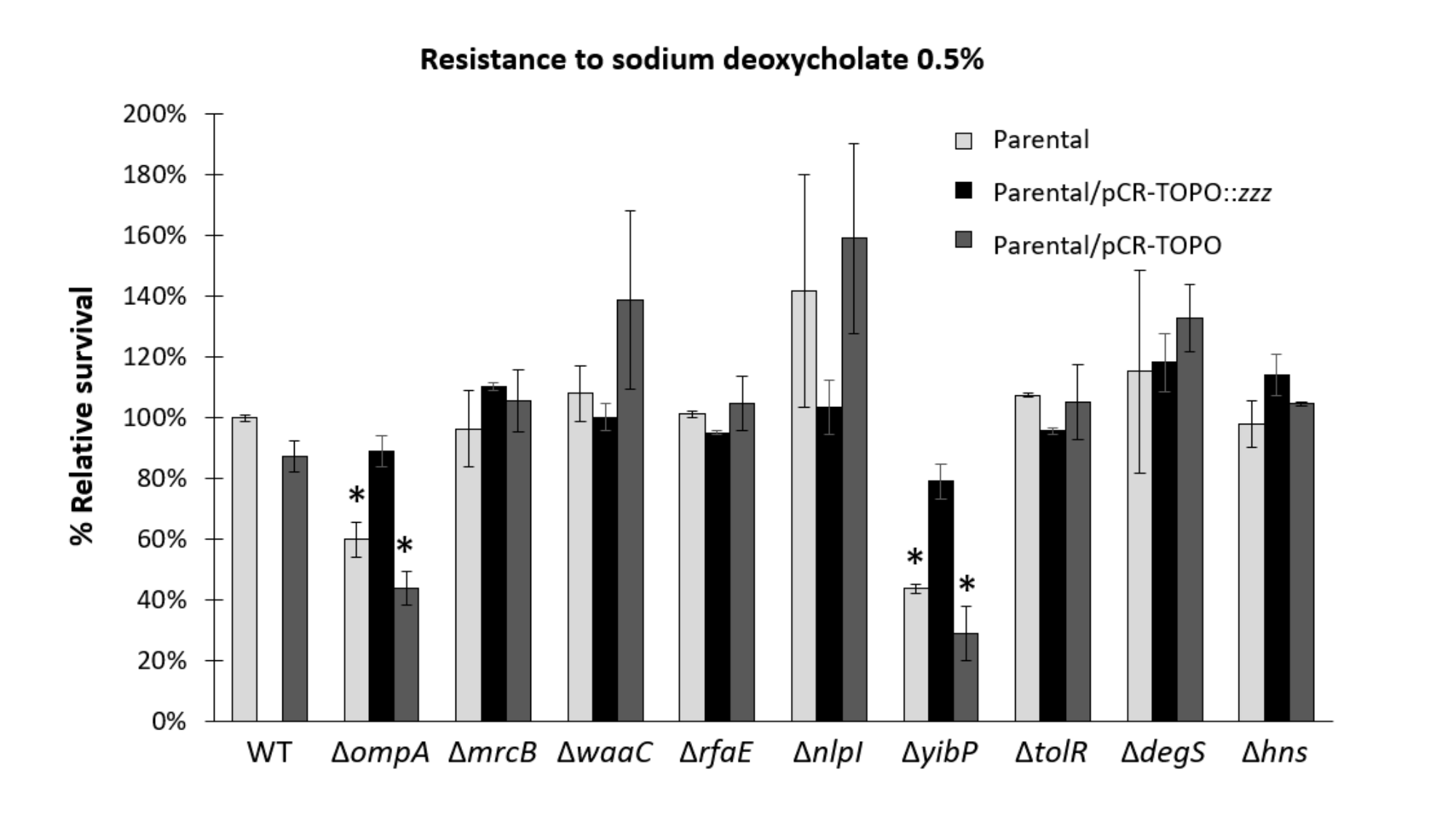

## Slide 4
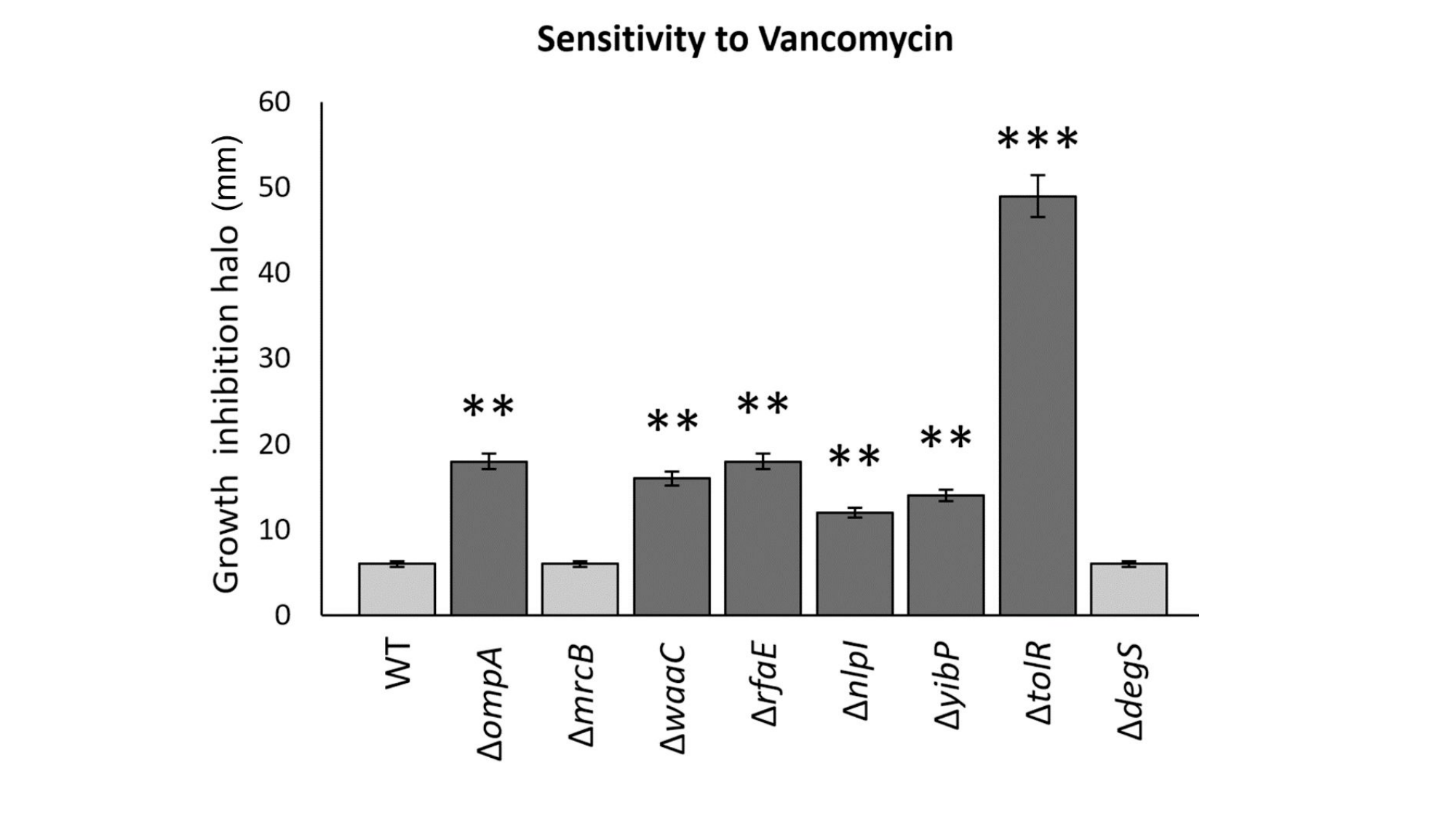

## Slide 5
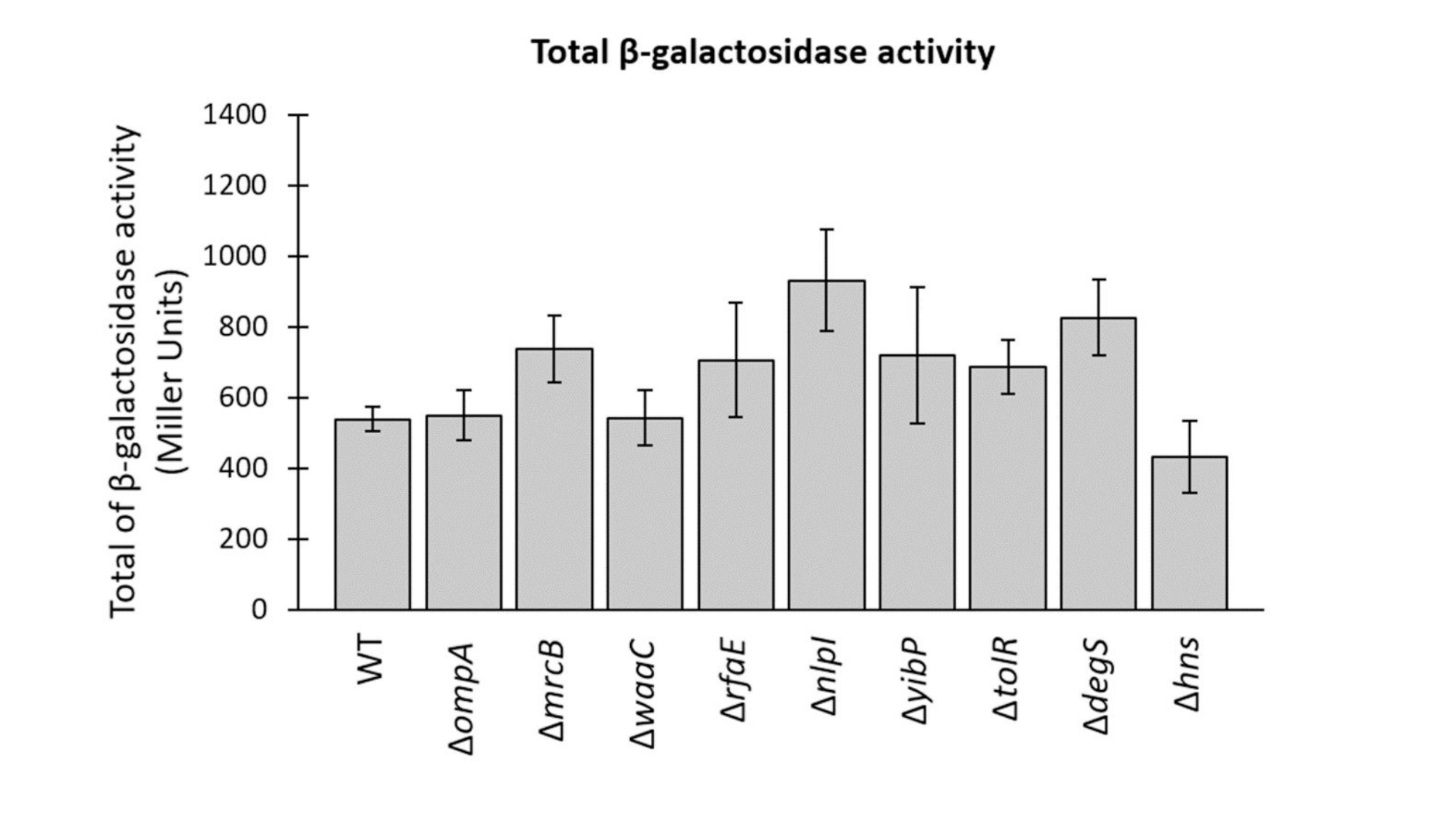

## Slide 6
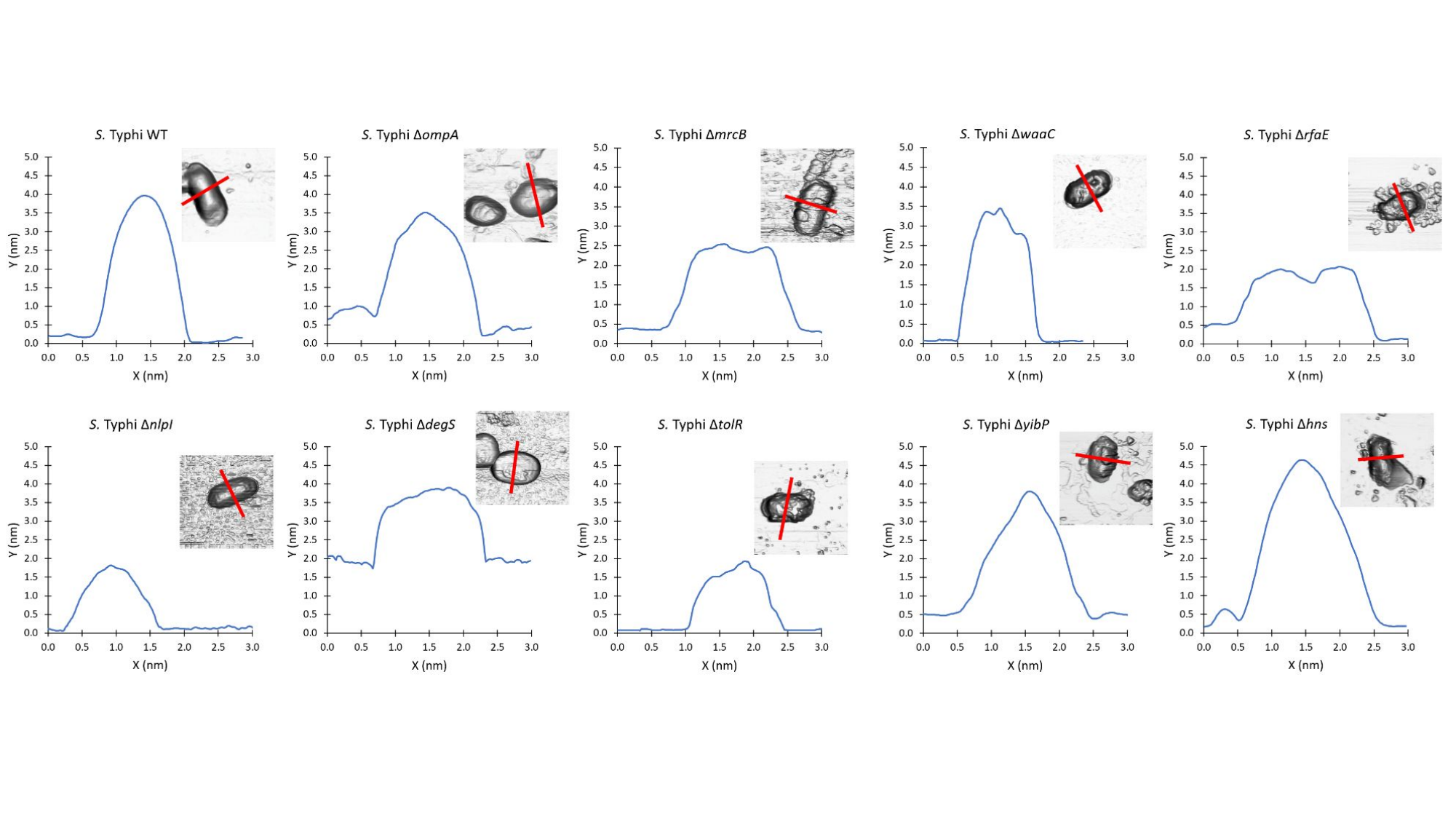

## Slide 7
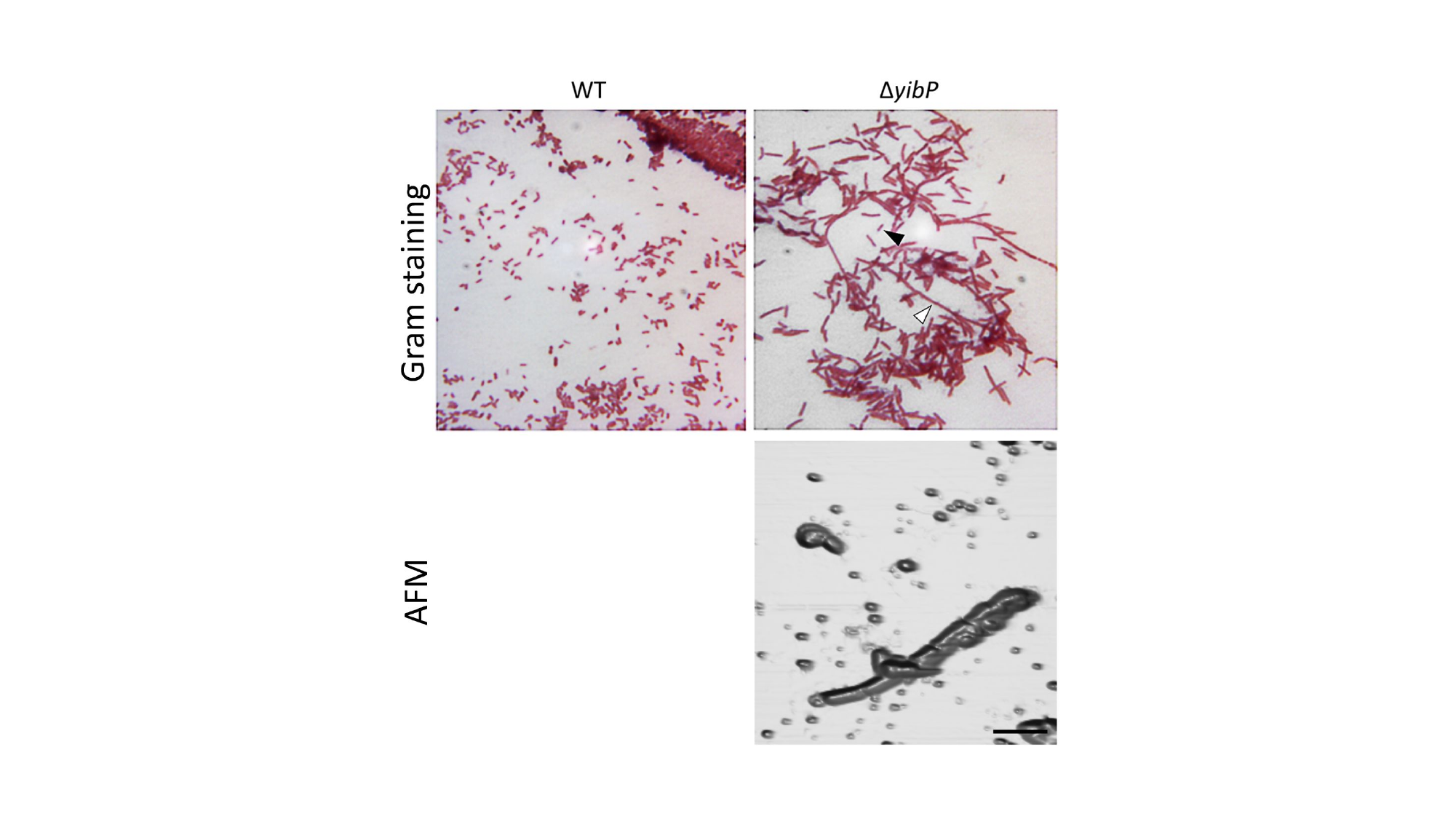

## Slide 8
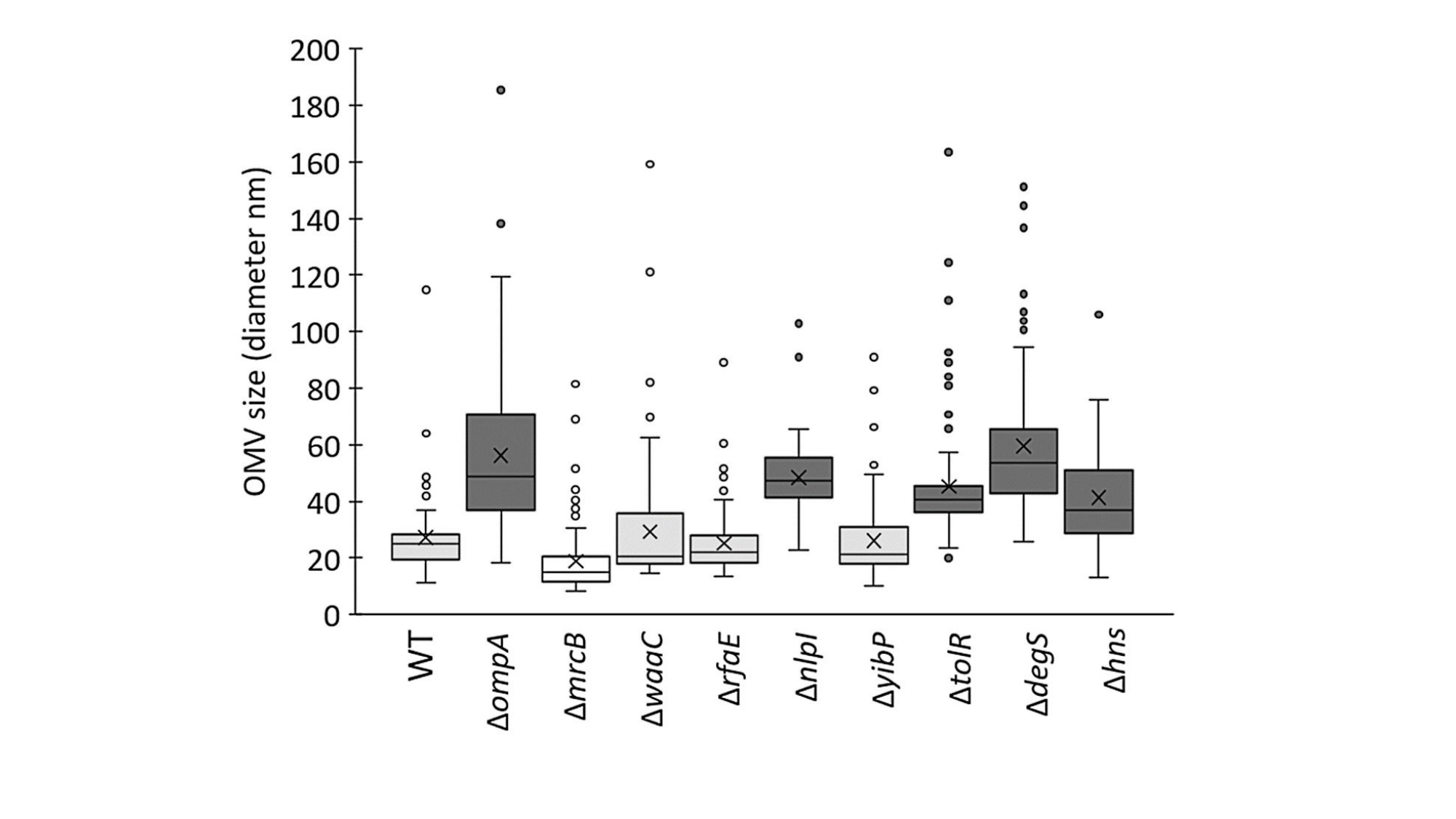

## Slide 9
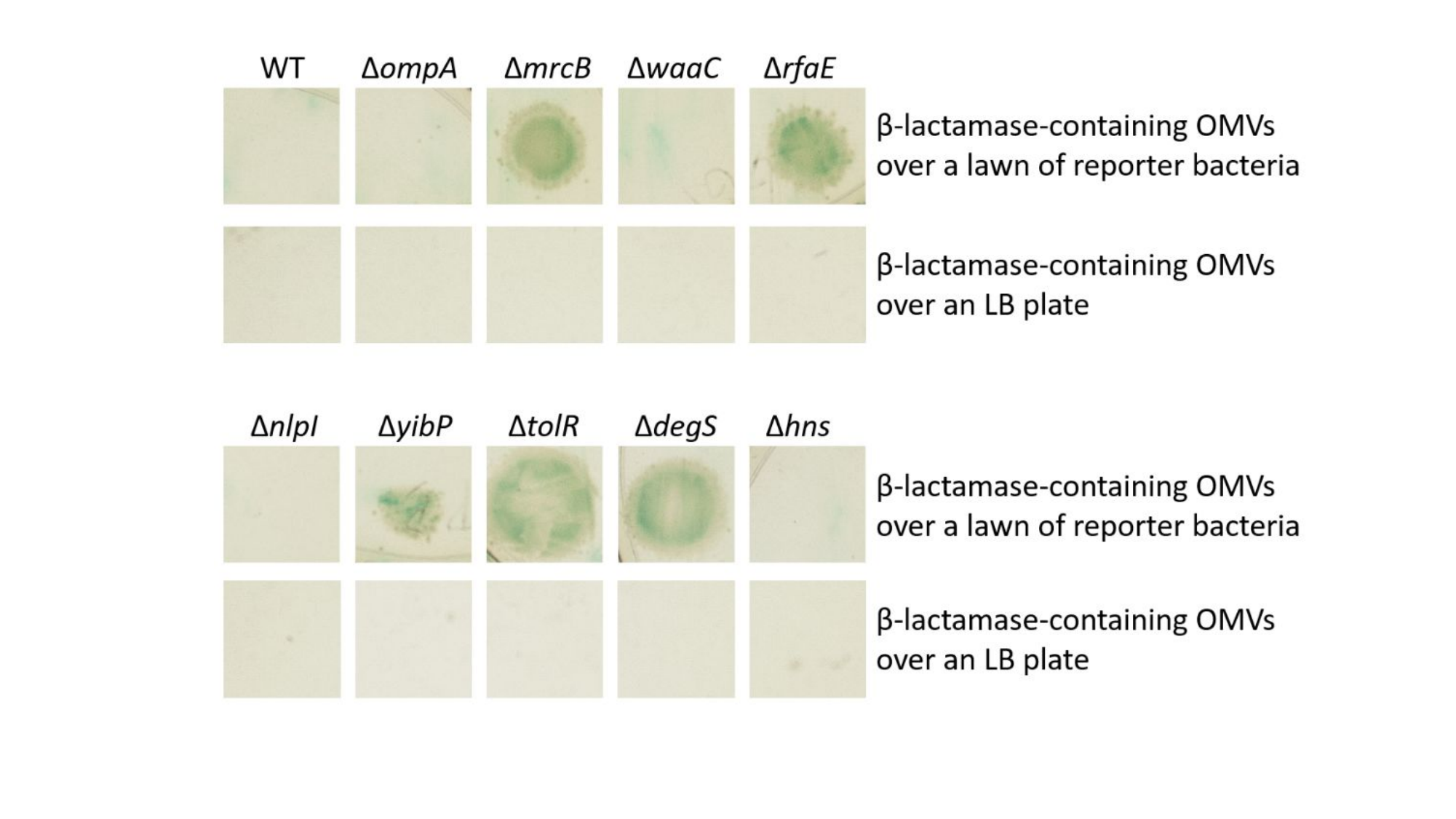

## Slide 10
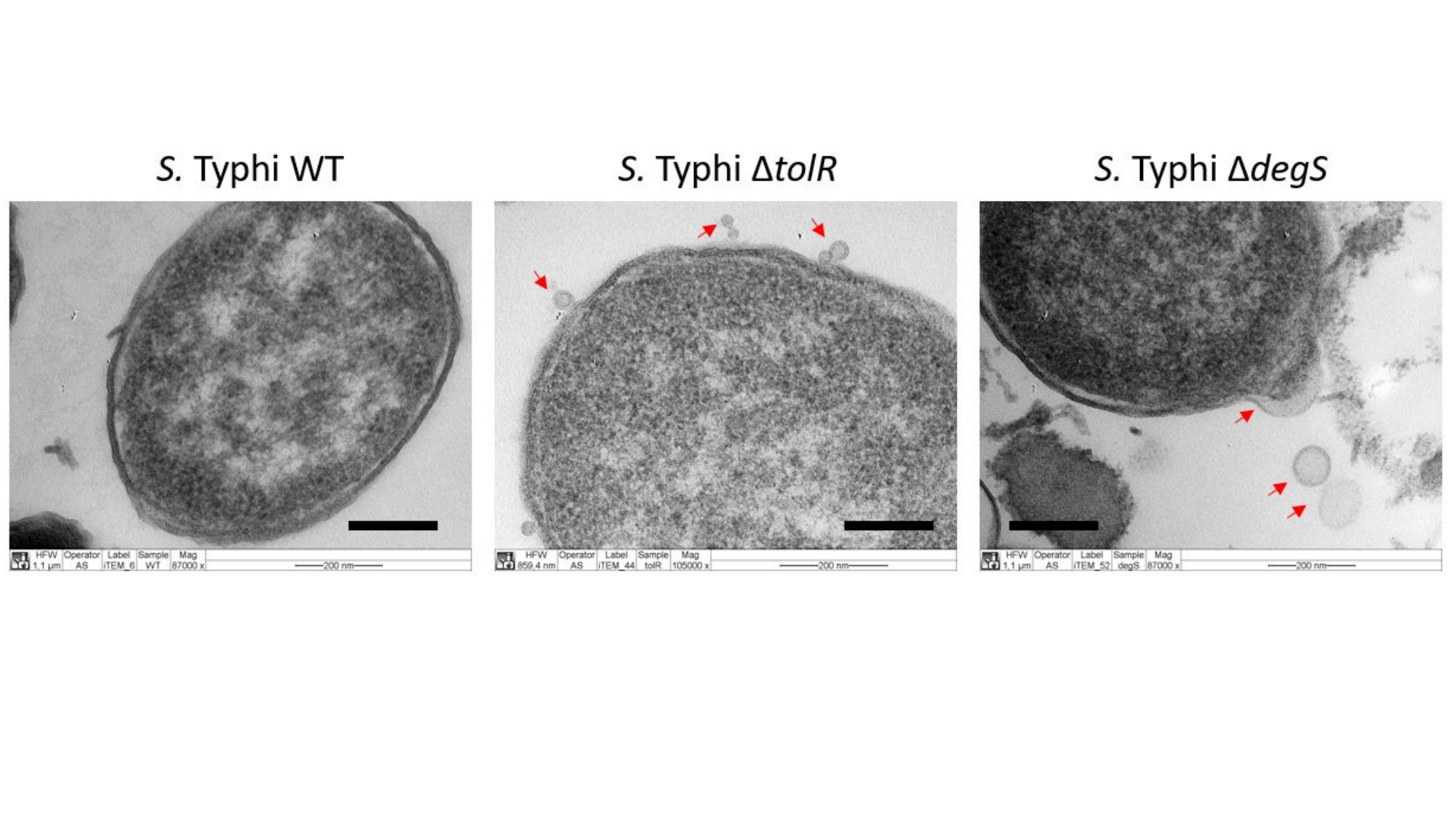

## Slide 11
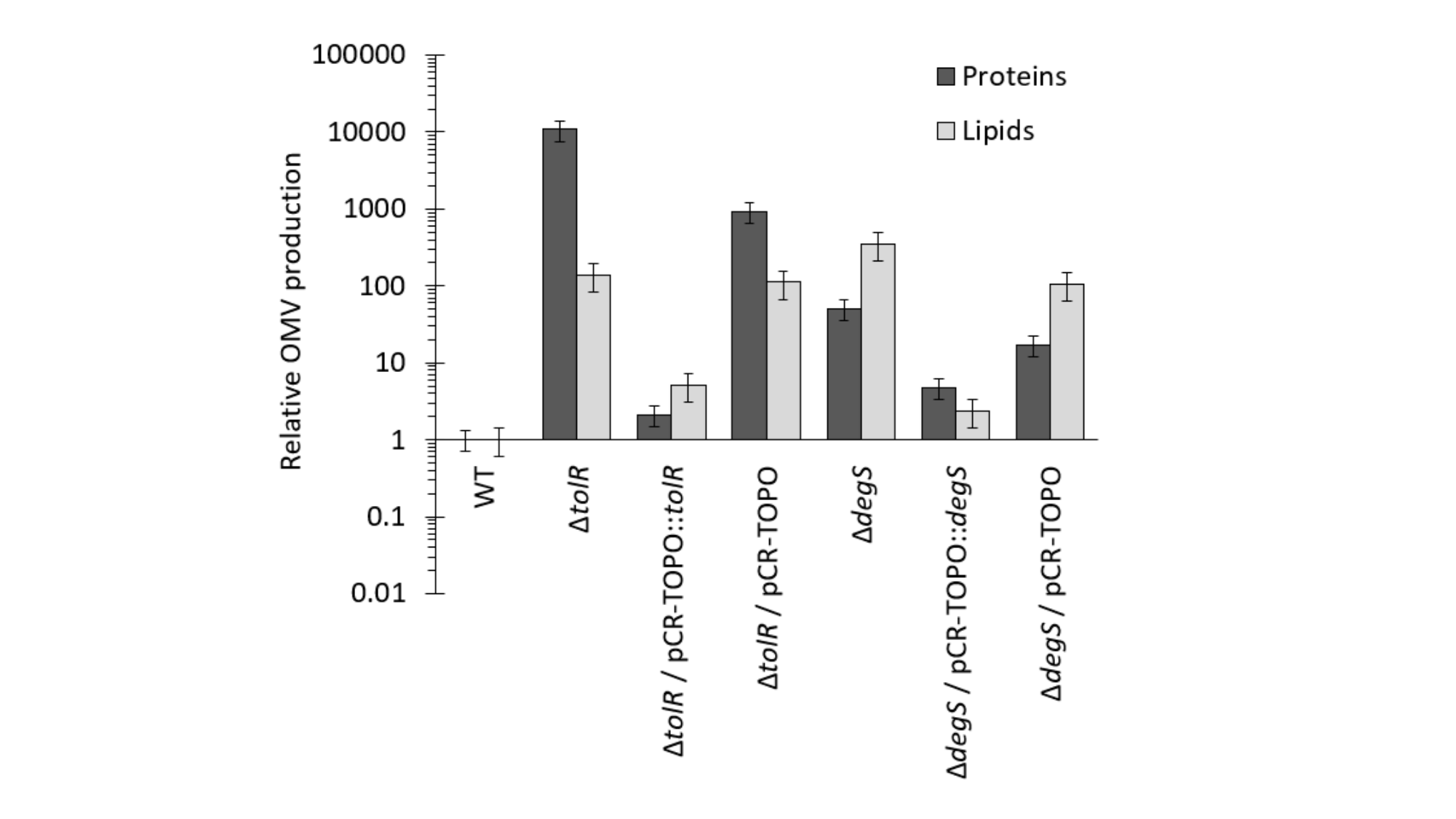

## Slide 12
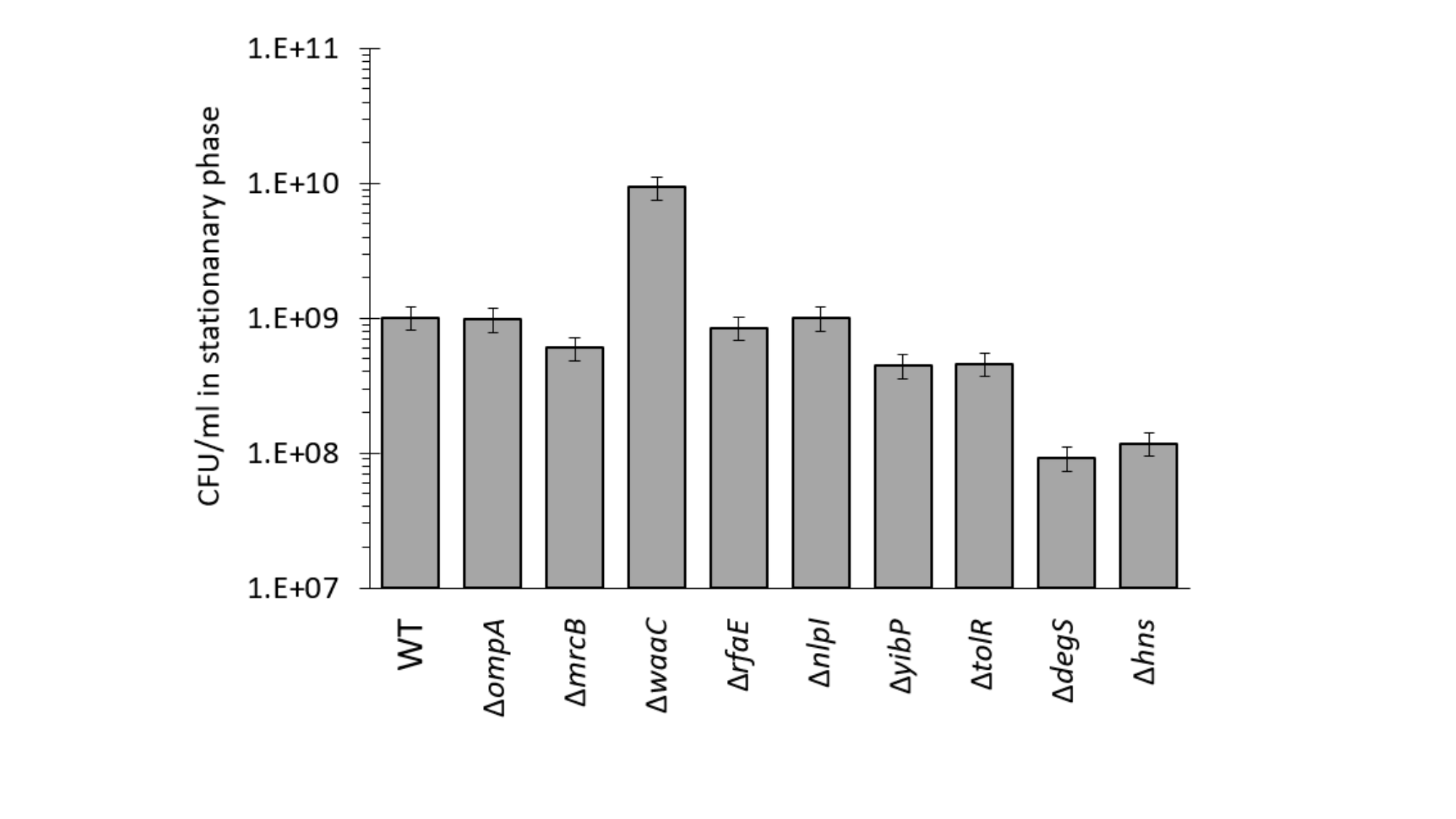

## Slide 13
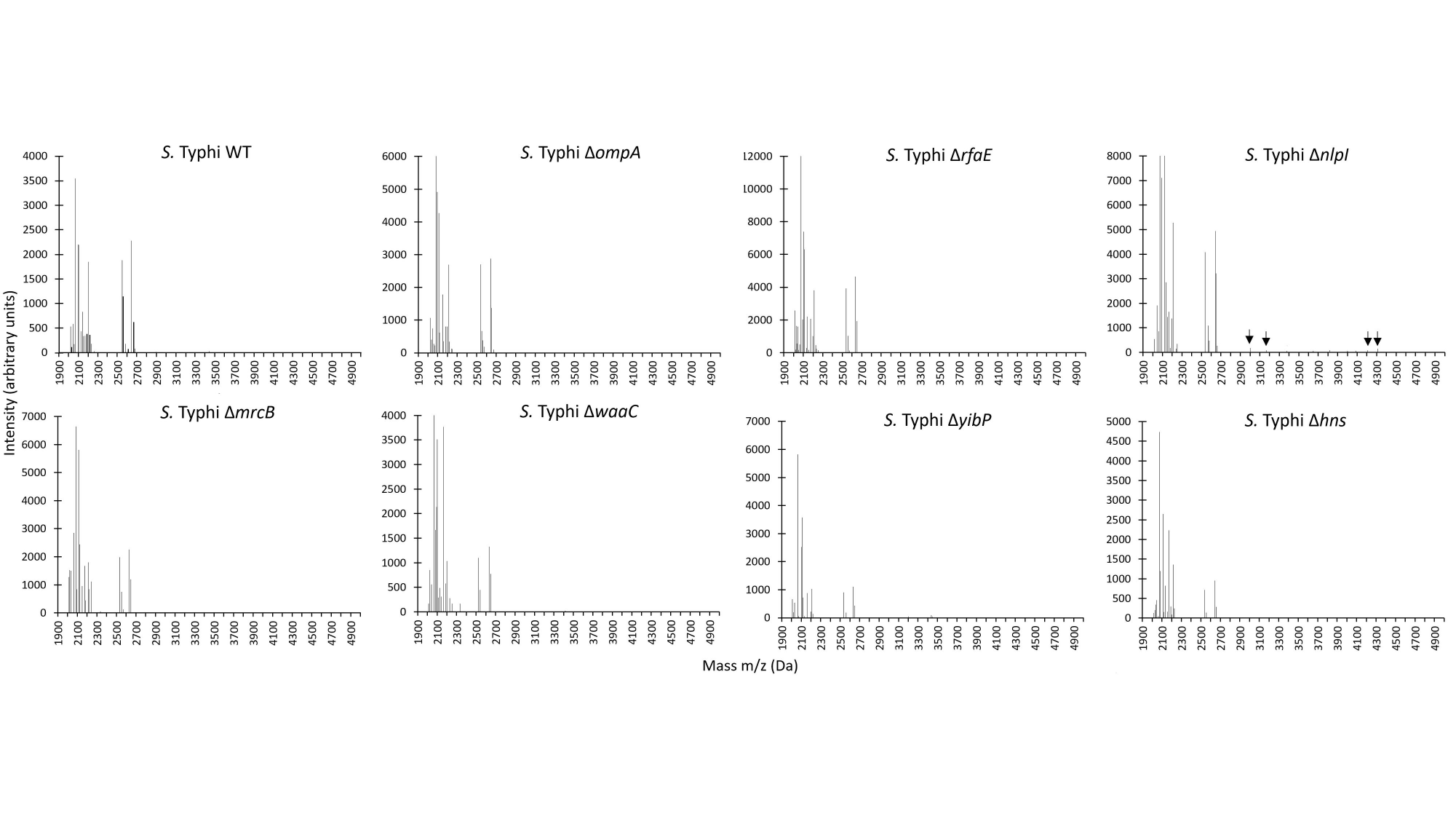

## Slide 14
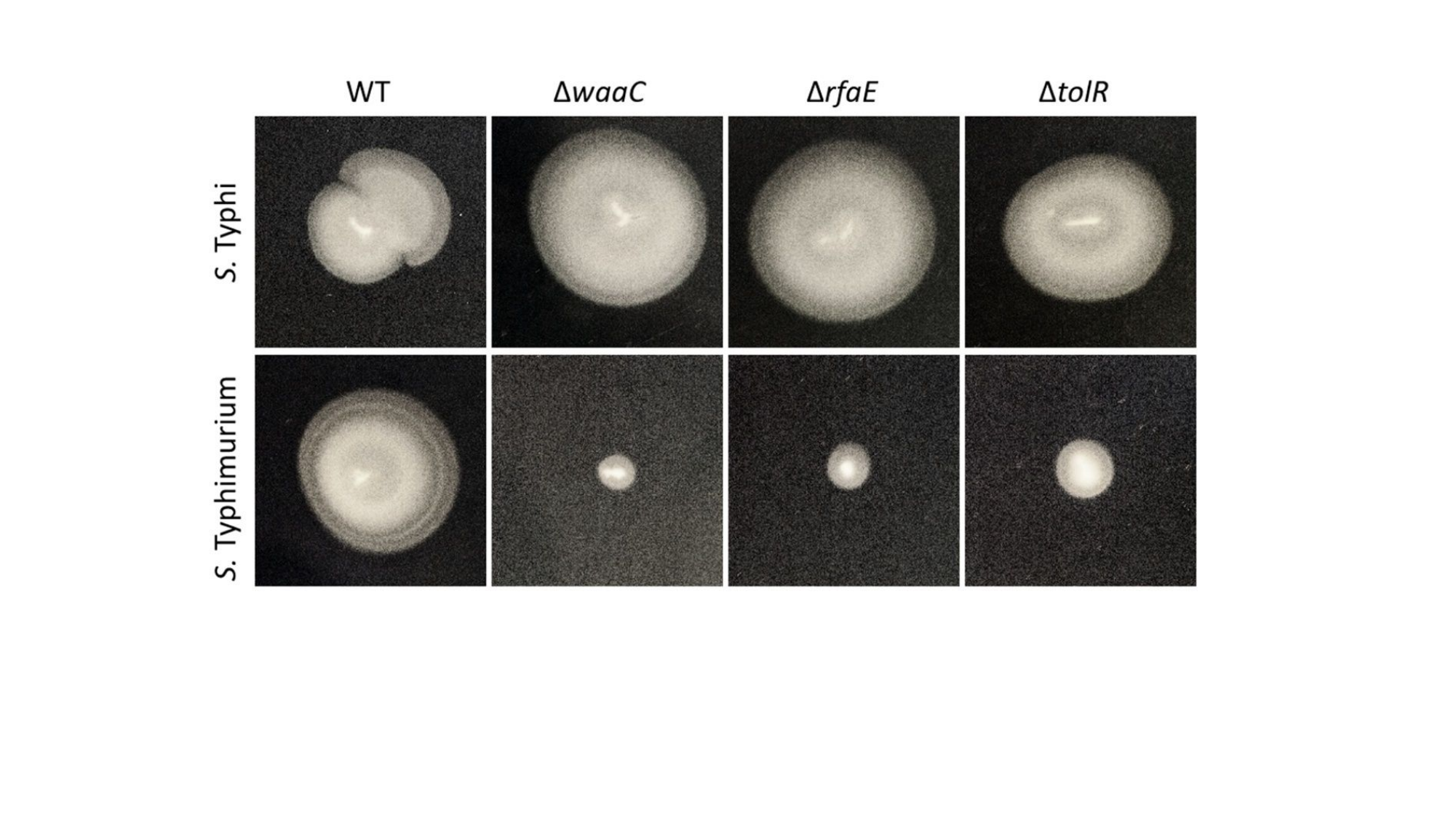

## Slide 15
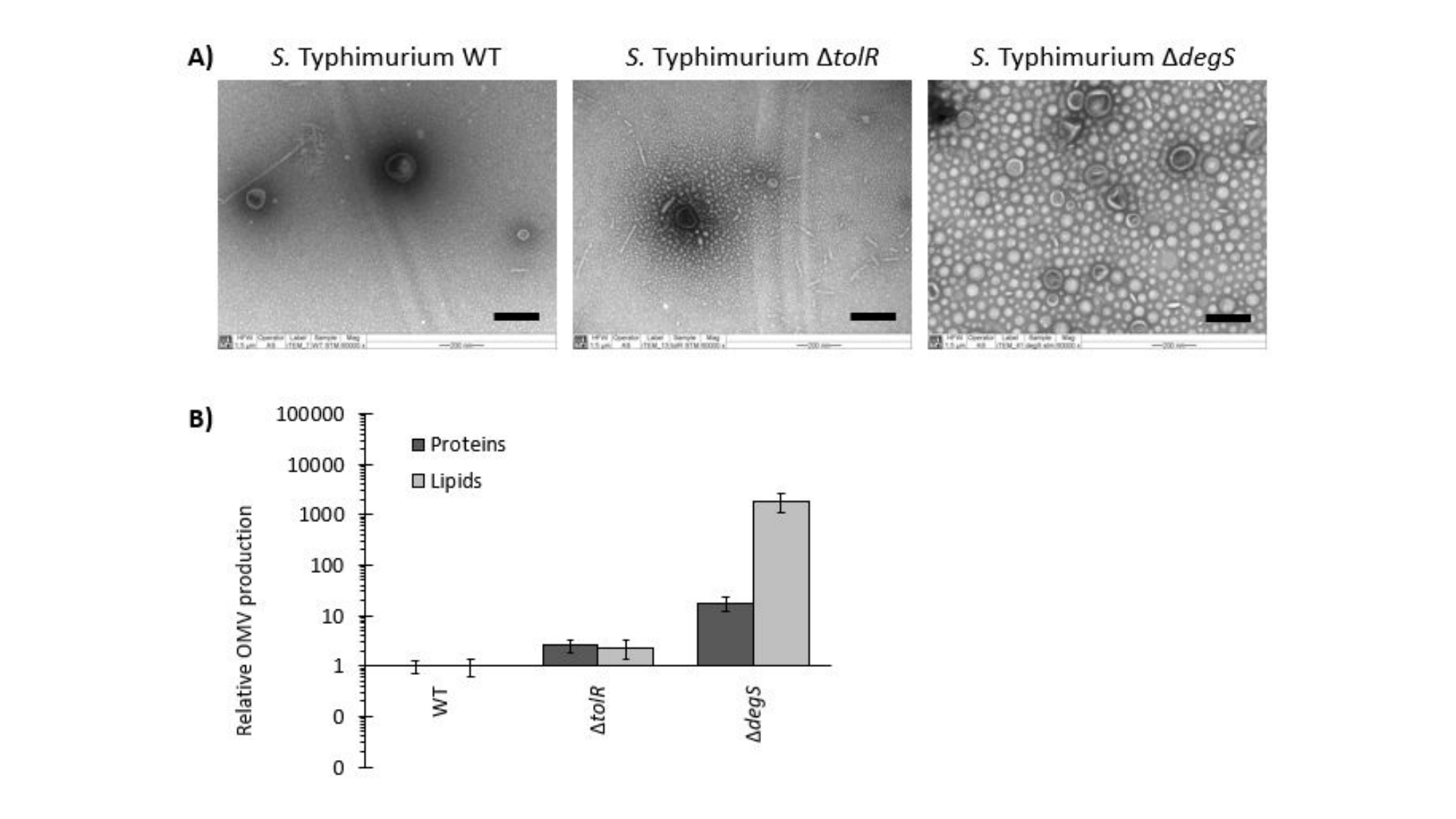

## Slide 16
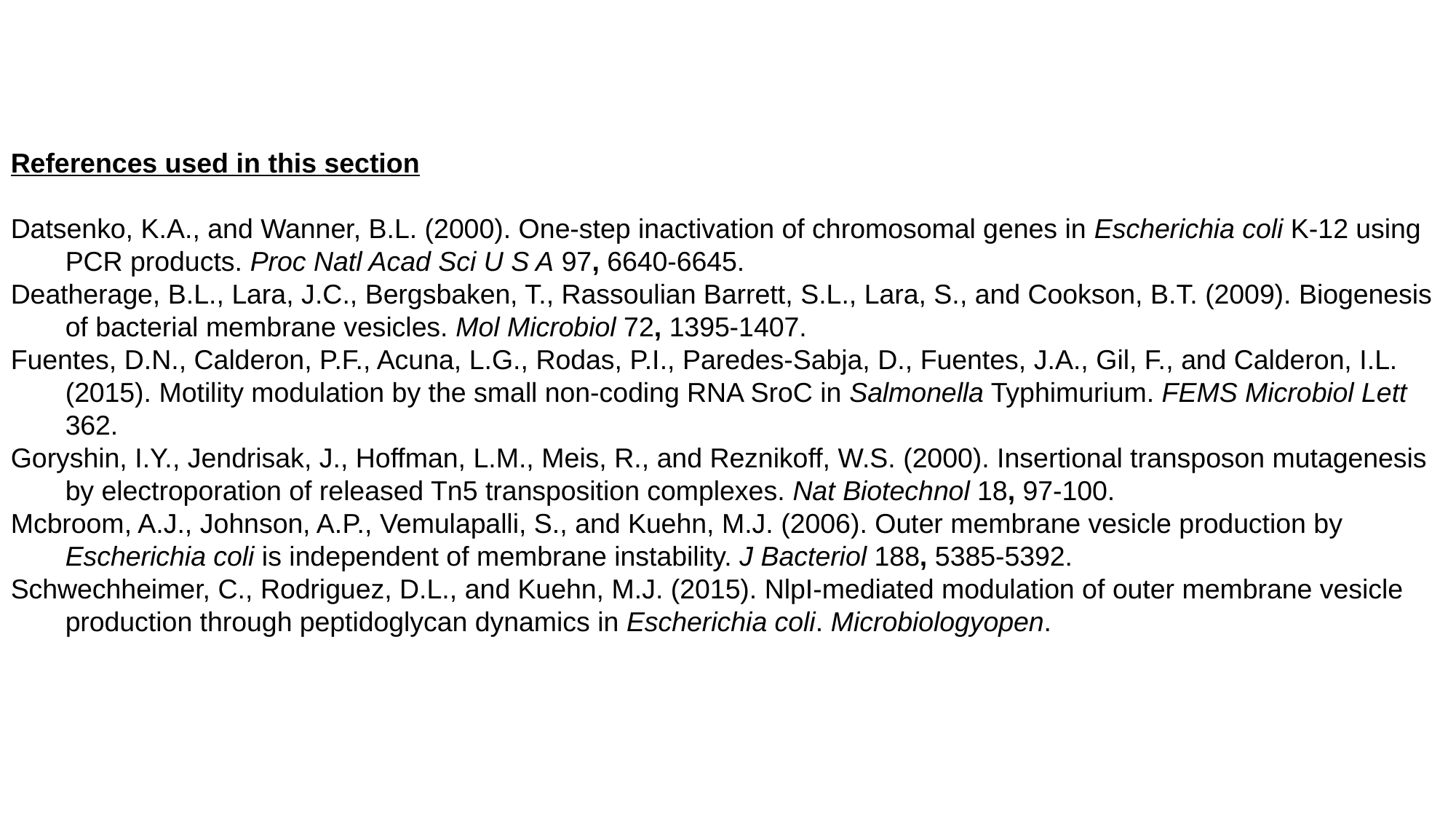

References used in this section
Datsenko, K.A., and Wanner, B.L. (2000). One-step inactivation of chromosomal genes in Escherichia coli K-12 using PCR products. Proc Natl Acad Sci U S A 97, 6640-6645.
Deatherage, B.L., Lara, J.C., Bergsbaken, T., Rassoulian Barrett, S.L., Lara, S., and Cookson, B.T. (2009). Biogenesis of bacterial membrane vesicles. Mol Microbiol 72, 1395-1407.
Fuentes, D.N., Calderon, P.F., Acuna, L.G., Rodas, P.I., Paredes-Sabja, D., Fuentes, J.A., Gil, F., and Calderon, I.L. (2015). Motility modulation by the small non-coding RNA SroC in Salmonella Typhimurium. FEMS Microbiol Lett 362.
Goryshin, I.Y., Jendrisak, J., Hoffman, L.M., Meis, R., and Reznikoff, W.S. (2000). Insertional transposon mutagenesis by electroporation of released Tn5 transposition complexes. Nat Biotechnol 18, 97-100.
Mcbroom, A.J., Johnson, A.P., Vemulapalli, S., and Kuehn, M.J. (2006). Outer membrane vesicle production by Escherichia coli is independent of membrane instability. J Bacteriol 188, 5385-5392.
Schwechheimer, C., Rodriguez, D.L., and Kuehn, M.J. (2015). NlpI-mediated modulation of outer membrane vesicle production through peptidoglycan dynamics in Escherichia coli. Microbiologyopen.
