# Supplementary material for: Identification of Genes Involved in Biogenesis of Outer Membrane Vesicles (OMVs) in Salmonella enterica Serovar Typhi
Source: Front Microbiol. 2019 Feb 4;10:104. doi: 10.3389/fmicb.2019.00104 (PMC6369716; doi:10.3389/fmicb.2019.00104)
Supplement: Supplementary file 2 [file Table_1.DOCX]

**Supplementary tables**

**Supp. Table 1.** Primers used in this study

| **Primers used to perform Red-Swap recombination** | |
| --- | --- |
| **Primer** | **Sequence** |
| ompA (H1+P1) | TAACGAGGCGCAAAAAATGAAAAAGACAGCTATCGCGATTTGTAGGCTGGAGCTGCTTCG |
| ompA (H2+P2) | TTTATCAGACGGAAACTTAAGCCTGCGGCTGAGTTACCACCATATGAATATCCTCCTTAG |
| mrcB (H1+P1) | AGAATCGGGCCTTTGCGCCTGTATGTTGCGGAGAAAAAGCTGTAGGCTGGAGCTGCTTCG |
| mrcB (H2+P2) | GCACTATTACCGTGATTAATTGCCGCCGAACATCTCCTTACATATGAATATCCTCCTTAG |
| waaC (H1+P1) | GGGTTTGCACAAATTCCGTCCTGGCACAGTGCTGTCGATCTGTAGGCTGGAGCTGCTTCG |
| waaC (H2+P2) | GGATGAGACAGAGTCTCTTTAATGAATCTTTCCAAATACGCATATGAATATCCTCCTTAG |
| rfaE (H1+P1) | GCAAATTTTGAATCTCTCAGGAGACAGGAATGAAAGTAAATGTAGGCTGGAGCTGCTTCG |
| rfaE (H2+P2) | GGTCTGGATCTTTTTGATGATATTGGTCGTGGAACAACCACATATGAATATCCTCCTTAG |
| nlpI (H1+P1) | TGTTTCGTTGCGACAGCACTTACGCTTGCAGGATGCAGTATGTAGGCTGGAGCTGCTTCG |
| nlpI (H2+P2) | TGTTCGTCAGCTATTGCTGGTCCGATTCTGCCAGGTCATCCATATGAATATCCTCCTTAG |
| yibP (H1+P1) | GCATTCAGCTTATTCTTAGCGGAGAAGAAAGCCAGCGTGGTGTAGGCTGGAGCTGCTTCG |
| yibP (H2+P2) | CTTTACCATGCTCTACGACCACCACCAGGCCATAGCCTTGCATATGAATATCCTCCTTAG |
| tolR (H1+P1) | CTGCTCGACGTACTGTTGGTGCTGCTGCTGATCTTTATGGTGTAGGCTGGAGCTGCTTCG |
| tolR (H2+P2) | GTTGATCCATTCGATCTTTATCAACCACTACGCTGTATTGCATATGAATATCCTCCTTAG |
| degS (H1+P1) | CCTTTTTTAACGACGCCTCCATCATGTTTGTGAAGCTCTTTGTAGGCTGGAGCTGCTTCG |
| degS (H2+P2) | GTATTCCTGCACCGTCACCTGGAACGTGAGCTGCTTATCACATATGAATATCCTCCTTAG |
| hns (H1+P1 | TATTAGCTCAACAAACCACCCCAATATAAGTTTGAGATTATGTAGGCTGGAGCTGCTTCG |
| hns (H2+P2) | GGATTTTAAGCATCCAGGAAGTAAATTATTCCTTGATCAGCATATGAATATCCTCCTTAG |
| fskK (H1+P1) | GCCGTAGTTTGATTACACTCCTGTTAATCCATACAGCAACTGTAGGCTGGAGCTGCTTCG |
| fskK (H2+P2) | GATCAGCTCTTCAACTTTTTTACCGACGGTCATCATCAGGCATATGAATATCCTCCTTAG |
| rfbH(H1+P1) | ATGACAGCAAATAACCTGCGTGAGCAAATCTCTCAGCTTGTGTAGGCTGGAGCTGCTTCG |
| rfbH(H2+P2) | TTAGAAATTCAAACCAAAGAACTCTTCAAACTTGCTAACTCATATGAATATCCTCCTTAG |
| ynhG (H1+P1) | CCATGTTAAGCATTAAAGATTTACAGGTCAGTGTGGAAGATGTAGGCTGGAGCTGCTTCG |
| ynhG (H2+P2) | GCTCGACAGAGAATTTCACTGGCTGGTTAATCACTTTTACCATATGAATATCCTCCTTAG |
| hns (H1+P1) | TATTAGCTCAACAAACCACCCCAATATAAGTTTGAGATTATGTAGGCTGGAGCTGCTTCG |
| hns (H2+P2) | GGATTTTAAGCATCCAGGAAGTAAATTATTCCTTGATCAGCATATGAATATCCTCCTTAG |
|  |  |
| **External primers used to corroborate Red-Swap Recombination** | |
| **Primer** | **Sequence** |
| ompA-N | TGCGAGAACGCTTGCCAGAAGC |
| ompA-C2 | TGGTCTGGCAGCGTCTGGCG |
| mrcB-N | CGTGACGTGTTATACGTTGCCT |
| mrcB-C | GCTGCGGTTACGGTAATGGTT |
| waaC-N | TTATCGCGTATGCCGGAAGT |
| waaC-C | ACTTTTCGGTGAGCGTGAGA |
| rfaE-N | CTTCTGGCGCAAAACGGCATT |
| rfaE-C | TGACCACGCCGTTGGTTTCAT |
| nlpI-N | ACGCTGGCGTGCCAATTAAA |
| nlpI-C | CGTAACTGCACGTCATAACGCT |
| yibP-N | AAACGCCGGTAAAGCGGTTCA |
| yibp-C | TGTGCGGGCGATAGCCAAAAT |
| tolR-N | TGGAAACGCTGGAAACGCAT |
| tolR-C | CTGGCTTGGCGGTTTAGGAAT |
| degS-N | TGCCGAACTGCGTTCACGTAT |
| degS-C | AGACGTCGGCACCGAATGTT |
| hns-N | TTTGCCCTCCGCATGTCACTA |
| hns-C | TGCAGTCGACTTACCCGCATT |
| fskK-N | TTGGTTTCCGGCGATTTCGACT |
| fskK-C | TTCAGATTGCCGTTGCTGGCTT |
| rfbH-N | ATGAGCAACATGGCGTTGGT |
| rfbH-C | GGTGGCAGTGATGTTCCACAA |
| ynhG-N | AAACGCCGGTAAAGCGGTTCA |
| ynhG-C | TGTGCGGGCGATAGCCAAAAT |
|  |  |
| **Primers used for epitope tagging** | |
| **Primer** | **Sequence** |
| hlyE-3xFLAG | AAGACACGGTAAGAAGACGCTTTTCGAGGTTCCTGACGTCGACTACAAAGACCATGACGG |
| hlyE-kan | GAATGCGGAAATCACCCTCGACTACCAGCTTAACGCCTGACCATATGAATATCCTCCTTAG |
|  |  |
| **Divergent primers used to sequence *loci* interrupted by Tn5** | |
| **Primer** | **Sequence** |
| KAN-2 FP-1 | ACCTACAACAAAGCTCTCATCAACC |
| R6KAN-2 RP-1 | CTACCCTGTGGAACACCTACATCT |
|  |  |
| **Primers used for RT-PCR** | |
| **Primer** | **Sequence** |
| RT-HLYE-C2 | CGCTTCATTCAGTTTCTTGA |
| RT-HLYE-N | AAGTTTTGCTTATGGACAGC |
| 16sFW | GTAGAATTCCAGGTGTAGCG |
| 16sRV | TTATCACTGGCAGTCTCCTT |

**Supp. Table 2.** OMV size (diameter in nm) and statistical significance

|  | | | **Mean difference (nm) and statistical significance** | | | | | | | | | |
| --- | --- | --- | --- | --- | --- | --- | --- | --- | --- | --- | --- | --- |
|  | **Mean ± SE (nm)** | **n** | **WT** | **Δ*ompA*** | **Δ*mrcB*** | **Δ*waaC*** | **Δ*rfaE*** | **Δ*nlpI*** | **Δ*yibP*** | **Δ*tolR*** | **Δ*degS*** | **Δ*hns*** |
| **WT** | 27.20 ± 1.56 | 77 | 0.00  ns | -29.08  **** | 8.35  ** | -2.09  ns | 2.05  ns | -21.18  **** | 1.05  ns | -18.11  **** | -32.27  **** | -14.04  **** |
| **Δ*ompA*** | 56.28 ± 2.74 | 101 |  | 0.00  ns | 37.43  **** | 26.99  **** | 31.13  **** | 7.90  ns | 30.13  **** | 10.97  ns | -3.19  ns | 15.03  ns |
| **Δ*mrcB*** | 18.85 ± 1.19 | 101 |  |  | 0.00  ns | -10.44  *** | -6.30  * | -29.53  **** | -7.30  ** | -26.46  **** | -40.62  **** | -22.40  **** |
| **Δ*waaC*** | 29.29 ± 2.09 | 101 |  |  |  | 0.00  ns | 4.14  ns | -19.09  **** | 3.13  ns | -16.02  **** | -30.18  **** | -11.96  **** |
| **Δ*rfaE*** | 25.15 ± 1.06 | 101 |  |  |  |  | 0.00  ns | -23.23  **** | -1.00  ns | -20.16  **** | -34.32  **** | -16.09  **** |
| **Δ*nlpI*** | 48.38 ± 1.31 | 101 |  |  |  |  |  | 0.00  ns | 22.23  **** | 3.07  ns | -11.09  ns | 7.14  ns |
| **Δ*yibP*** | 26.16 ± 1.30 | 101 |  |  |  |  |  |  | 0.00  ns | -19.16  **** | -33.32  **** | -15.09  **** |
| **Δ*tolR*** | 45.31 ± 2.07 | 101 |  |  |  |  |  |  |  | 0.00  ns | -14.16  * | 4.07  ns |
| **Δ*degS*** | 59.47 ± 2.58 | 101 |  |  |  |  |  |  |  |  | 0.00  ns | 18.23  ** |
| **Δ*hns*** | 41.25 ± 2.08 | 66 |  |  |  |  |  |  |  |  |  | 0.00  ns |

Statistical test: Kruskal-Wallis with Dunn’s multiple comparison test.

SE: Standard error of mean

ns: Not significant differences; * *p* < 0.5; ** *p* < 0.1; *** *p* < 0.001; **** *p* < 0.0001
